# Unravelling the Chemolithoautotrophic Metabolism of the Carboxydotrophic *Knallgas* Bacterium *Hydrogenophaga pseudoflava*

**DOI:** 10.1111/1751-7915.70452

**Published:** 2026-11-01

**Authors:** Simon Grieshaber, Antonia Ebert, Pauline Kehr, Dominik L. Siebert, Ammar Al‐Shameri, Volker Sieber, Birgitta E. Ebert, Esteban Marcellin, Bastian Blombach

**Affiliations:** ^1^ Microbial Biotechnology, Campus Straubing for Biotechnology and Sustainability Technical University of Munich Straubing Germany; ^2^ Australian Institute for Bioengineering and Nanotechnology The University of Queensland Brisbane Queensland Australia; ^3^ ARC Centre of Excellence in Synthetic Biology The University of Queensland Brisbane Queensland Australia; ^4^ Advanced Engineering Biology Future Science Platform CSIRO Brisbane Queensland Australia; ^5^ Chair of Chemistry of Biogenic Resources, Campus Straubing for Biotechnology and Sustainability Technical University of Munich Straubing Germany; ^6^ Catalytic Research Center Technical University of Munich Garching Germany; ^7^ SynBiofoundry@TUM Technical University of Munich Straubing Germany

**Keywords:** aerobic gas fermentation, branched respiratory chain, carbon dioxide, carbon monoxide, chemolithoautotrophic metabolism, CO dehydrogenase, hydrogenase, *Hydrogenophaga pseudoflava*, proteomics, transhydrogenase

## Abstract

The sustainable production of chemicals is critical to reducing greenhouse gas emissions and environmental pollution. Gaseous feedstocks from industrial waste streams and waste gasification, including CO, CO_2_ and H_2_, represent abundant and underutilized resources for biotechnology. Syngas fermentation has been predominantly studied under anaerobic conditions, whereas aerobic processes may offer advantages for the biosynthesis of energy‐intensive products. One promising organism is the aerobic carboxydotroph *Hydrogenophaga pseudoflava* DSM 1084, whose chemolithoautotrophic metabolism remains poorly understood. To address this, we performed proteome analysis under different growth conditions and characterized deletion mutants of key enzymes involved in CO and H_2_ oxidation. Deletion mutants showed that only CO dehydrogenase I and the membrane‐bound hydrogenase are essential for CO and H_2_ oxidation, respectively. The role of the putative second CO dehydrogenase remains to be elucidated, and the soluble hydrogenase may contribute to redox balancing under microaerobic or anaerobic conditions. In addition, the H_2_‐sensing regulatory system appears to be nonfunctional, and the transhydrogenase PntAB supports redox homeostasis during chemolithoautotrophic growth. Electrons from CO or H_2_ are transferred via the reduced quinone pool to NADH through proton‐motive force‐driven reverse electron transfer. Nevertheless, growth on CO remains slower than on CO_2_/H_2_. In contrast to CO_2_/H_2_, an unphysiologically high CO concentration (80%) led to a major proteome rearrangement, with 1879 of 3640 reliably quantified proteins showing altered abundance compared to fructose‐grown cells, including a pronounced stress response. Under such conditions, cells are likely to invest additional energy to maintain redox balance. Together with the less energy‐conserving autotrophic branch of the respiratory chain and cytoplasmic CO oxidation, this results in inefficient CO conversion and reduced growth.

## Graphical abstract

Proteomics of the aerobic carboxydotroph *Hydrogenophaga pseudoflava* across trophic conditions revealed that an unphysiological CO concentration causes a redox imbalance and stress response. CO dehydrogenase I and the membrane‐bound hydrogenase were essential for CO and H_2_ oxidation, respectively, whereas the soluble hydrogenase likely supports redox balance, and the H_2_‐sensing regulatory system appears to be nonfunctional. Furthermore, the transhydrogenase PntAB is involved in redox homeostasis during chemolithoautotrophic growth.

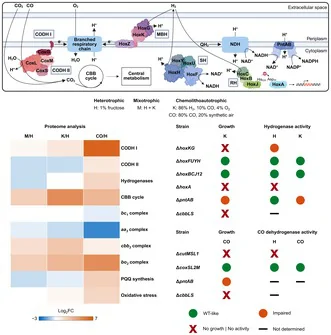

## 1 Introduction

To advance the sustainability of biotechnological processes, it is essential to use alternative carbon and energy sources that do not compete with the food and feed industry (Kiefer et al. 2021). Gas fermentation offers a promising alternative, as it enables the conversion of gaseous industrial side streams containing carbon monoxide (CO), carbon dioxide (CO_2_) and hydrogen (H_2_), which is referred to as synthesis gas or syngas (Dürre and Eikmanns 2015). Alternatively, syngas can be generated through gasification or pyrolysis of biomass or (organic) waste residues (Liew et al. 2016). Anaerobic gas fermentation of syngas derived from steel mills with acetogenic bacteria has already demonstrated technological feasibility at an industrial scale, highlighting its potential for sustainable production of chemicals and fuels (Neto et al. 2025). However, expanding the product spectrum from short‐chain chemicals such as ethanol or acetate to longer‐chain and ATP‐intensive products is hindered by thermodynamic constraints due to the anaerobic lifestyle of these bacteria (Takors et al. 2018). In contrast, aerobic oxidation of CO using molecular oxygen (O_2_) as terminal electron acceptor yields more energy than anaerobic CO oxidation by acetogenic bacteria (ΔG°′ = −257 vs. −44.4 kJ mol^−1^) (Kohtz et al. 2026).

Therefore, a promising alternative is the application of aerobic carboxydotrophic *Knallgas* bacteria, which naturally oxidize CO or H_2_ to enable CO_2_ fixation (Siebert et al. 2021). *Hydrogenophaga pseudoflava* DSM 1084 has gained potential as a production host, as it grows comparably fast under chemolithoautotrophic conditions with CO, CO_2_/H_2_, or mixtures thereof (Cypionka et al. 1980; Grenz et al. 2019; Miserez et al. 2026). Furthermore, this Gram‐negative β‐proteobacterium tolerates CO concentrations up to 90% (v v^−1^) (Cypionka and Meyer 1982). Grenz et al. (2019) sequenced and annotated its genome and developed a genetic engineering toolbox that enables markerless chromosomal modifications and inducible expression of target genes. These tools were applied to engineer *H. pseudoflava* for the aerobic production of the C_15_ sesquiterpene (E)‐α‐bisabolene from syngas (Grenz et al. 2019). Furthermore, Miserez et al. (2026) optimized the gas feed and media composition and improved *H. pseudoflava* growth on syngas, achieving cell dry weight (CDW) concentrations of over 11 g L^−1^.

The key enzyme for utilizing CO as an energy source is the O_2_‐insensitive CO dehydrogenase (CODH), which is highly conserved in all aerobic carboxydotrophic bacteria (Meyer and Schlegel 1983; Siebert et al. 2021). It consists of three subunits: the large subunit contains a molybdopterin binding site, the medium subunit a FAD‐binding site, and the small subunit has two [2Fe‐2S] center‐binding sites (Kang and Kim 1999). Its primary functions lie in supplying electrons to the respiratory chain, generating ATP via oxidative phosphorylation and NADH through reverse electron transfer (RET), and providing CO_2_ as a carbon source, which is subsequently fixed through the Calvin–Benson–Bassham (CBB) cycle (Meyer and Schlegel 1983; Siebert et al. 2021). Two putative CODHs are annotated in *H. pseudoflava* (*cutMSL1* and *coxSL2M*), belonging to the OMP (after *Oligotropha*, *Mycobacterium* and *Pseudomonas*) and BMS (after *Bradyrhizobium*, *Mesorhizobium* and *Sinorhizobium*) groups, respectively (Grenz et al. 2019; Siebert et al. 2021). Both types are assumed to differ in their substrate affinity and to be differentially expressed depending on the environmental CO concentration (King 2003). CODH of the aerobic carboxydotrophic bacterium *Afipia carboxidovorans* OM5 reduces quinones rather than cytochromes (Wilcoxen et al. 2011), and recent work in *Mycobacterium smegmatis* mc^2^155 demonstrated electron transfer to the aerobic respiratory chain via the quinone‐binding protein CoxG (Kropp et al. 2025). A similar long‐range quinone transfer mechanism is likely operative in *H. pseudoflava* , which predominantly uses ubiquinone‐8 (Willems et al. 1989). Durante‐Rodríguez et al. (2024) characterized the transcriptional regulator CoxC of *A. carboxidovorans* OM5, which controls the expression of the *cox* genes encoding CODH. CoxC functions as a CO‐responsive repressor, in which the membrane‐bound MHYT domain senses CO via a copper‐dependent mechanism, leading to derepression of the target promoter (Durante‐Rodríguez et al. 2024). In *H. pseudoflava* , the genome annotation indicates a putative RcoM‐type regulator located upstream of the CODH I (*cutMSL1*) cluster (Grenz et al. 2019; Siebert et al. 2021), which likely senses CO through an N‐terminal heme‐bearing PAS fold regulatory domain rather than a membrane‐bound MHYT domain (Kerby et al. 2008; Dent et al. 2023). No regulatory gene is annotated upstream of the second CODH cluster (Grenz et al. 2019). Besides CO sensing, regulation is likely affected by carbon catabolite repression (Conrad et al. 1981; Cypionka and Meyer 1982; Dent et al. 2023).

Carboxydotrophic bacteria primarily utilize CO for energy conservation through oxidative phosphorylation and the generation of reducing equivalents, with only 4%–16% of the CO‐derived CO_2_ being assimilated via the CBB cycle (Zavarzin and Nozhevnikova 1977; Meyer and Schlegel 1978). The ability to utilize CO under aerobic conditions is enabled by a branched respiratory chain that diverges at the level of *b*‐type cytochromes or ubiquinone (Cypionka and Meyer 1983a). In *A. carboxidovorans* OM5, the autotrophic branch includes cytochrome *b*_*563*_ (cytochrome *o*), which functions as the CO‐insensitive terminal oxidase (Cypionka and Meyer 1983b). This oxidase shows a higher affinity for O_2_ than cytochrome *a*_*1*_ oxidase, the CO‐sensitive terminal oxidase of the heterotrophic branch (Cypionka et al. 1985). Yet, energy conservation within the CO‐insensitive branch is lower than in the CO‐sensitive counterpart (Cypionka et al. 1984). Because CODH cannot directly reduce NAD(P)^+^, carboxydotrophic *Knallgas* bacteria rely on proton‐motive force (PMF)‐driven RET to generate NAD(P)H (Meyer and Schlegel 1979; Jacobitz and Meyer 1986).

In addition to CO, *H. pseudoflava* can also utilize H_2_ as an energy source, as it possesses an O_2_‐tolerant membrane‐bound hydrogenase (MBH) (Cypionka et al. 1980). In *Cupriavidus necator* H16, this enzyme consists of a large subunit containing the [NiFe] active site, a small subunit harbouring three [Fe‐S] clusters, and a membrane‐integrated cytochrome *b* with two heme groups (Bernhard et al. 1997; Fritsch et al. 2011). The MBH of *A. carboxidovorans* OM5 similarly consists of three subunits (HoxSLZ) encoded on the megaplasmid pHCG3 (Santiago and Meyer 1997). Corresponding *hox* genes of *H. pseudoflava* are located on the chromosome (Grenz et al. 2019). Equally to CO oxidation, H_2_‐derived electrons from MBH enter the respiratory chain, contributing to PMF generation that drives ATP synthesis and the production of reducing equivalents via RET (Wheelis 1984). Moreover, the hydrogenase encoding cluster in *H. pseudoflava* shares a similar structural organization with that of *C. necator* H16 (Siebert et al. 2021). It also contains genes that encode a putative H_2_‐sensing transcriptional regulatory system and a soluble hydrogenase (SH) (Grenz et al. 2019). In vitro, the latter was recently shown to directly reduce NAD^+^ using H_2_ (Siebert et al. 2026). Unlike many other carboxydotrophic bacteria, *H. pseudoflava* exhibits hydrogenase activity even under heterotrophic growth conditions (Kiessling and Meyer 1982). Another key enzyme contributing to redox balance in *H. pseudoflava* is the membrane‐bound transhydrogenase PntAB (Grenz et al. 2019). This enzyme utilizes the proton gradient to drive the transfer of electrons from NADH to NADP^+^ (Pedersen et al. 2008). However, its role in aerobic carboxydotrophic bacteria remains largely uncharacterized.

Although fundamental knowledge of the biochemistry of CODH, MBH, and the branched respiratory chain has been gathered, the overall physiology and regulation of the chemolithoautotrophic metabolism in carboxydotrophic *Knallgas* bacteria remain poorly understood. To gain a holistic understanding of *H. pseudoflava* DSM 1084 metabolism and its adaptation to chemolithoautotrophy, we performed proteome analysis under heterotrophic, mixed‐substrate (hereafter referred to as mixotrophic) and chemolithoautotrophic growth conditions. For the first time, we constructed and analysed a set of mutant strains carrying deletions of structural genes encoding putative CODHs, hydrogenases and the transhydrogenase PntAB in a carboxydotrophic *Knallgas* bacterium.

## 2 Materials and Methods

### 2.1 Bacterial Strains, Plasmids and Media

All bacterial strains and plasmids used are listed in Table S1. The following complex media were used for cultivation (Atlas 2010; Green and Sambrook 2012): TB (5 g tryptone, 3 g beef extract L^−1^) and LB medium without NaCl (LBo; 10 g tryptone, 5 g yeast extract L^−1^) for *H. pseudoflava* and 2×TY (16 g tryptone, 10 g yeast extract, 5 g NaCl L^−1^) for *Escherichia coli* . As a minimal medium, the ‘carbon monoxide oxidizer medium’ (CO‐Ox), described by the Leibniz Institute DSMZ—German Collection of Microorganisms and Cell Cultures GmbH (DSMZ; Braunschweig, Germany), was used and contained: 1.78 g Na_2_HPO_4_, 1.50 g NH_4_Cl and 0.75 g KH_2_PO_4_ per litre. To buffer the medium, 10 g 3‐(N‐morpholino)propanesulfonic acid L^−1^ was added (Grenz et al. 2019), and the pH was adjusted to 7 with 5 N KOH. After autoclaving, the medium was supplemented with 0.20 g MgSO_4_ × 7 H_2_O, 0.03 g CaCl_2_ × 2 H_2_O, 0.018 g ammonium ferric citrate and 1 mL trace element solution SL‐6 (cf. DSMZ) per litre. The trace element solution contained: 0.10 g ZnSO_4_ × 7 H_2_O, 0.03 g MnCl_2_ × 4 H_2_O, 0.30 g H_3_BO_3_, 0.20 g CoCl_2_ × 6 H_2_O, 0.01 g CuCl_2_ × 2 H_2_O, 0.02 g NiCl_2_ × 6 H_2_O, 0.03 g Na_2_MoO_4_ × 2 H_2_O per litre. For nutrient‐reduced experiments, supplements were added at 10% of their respective standard concentrations described above. 18 g agar L^−1^ was added to the liquid medium for agar plates. 25 ( *H. pseudoflava* ) or 50 μg ( *E. coli* ) kanamycin mL^−1^ were supplemented when necessary. Plasmid‐based gene expression was induced by adding 1 mM isopropyl β‐𝒟‐1‐thiogalactopyranoside at the beginning of the main cultivation. For gas fermentation, two different gas mixtures, ordered premixed from Linde GmbH (Pullach, Germany), were used: a CO_2_/H_2_ gas mixture with 10% CO_2_, 86% H_2_ and 4% O_2_, and a CO gas mixture with 80% CO and 20% synthetic air (80% N_2_, 20% O_2_), resulting in a final gas composition of 80% CO, 16% N_2_ and 4% O_2_.

### 2.2 Cultivation Conditions and Growth Measurements

In general, all *H. pseudoflava* strains were cultivated at 30°C and 120 rpm, *E. coli* strains at 37°C and 180 rpm (Multitron Standard and Ecotron, Ø = 25 mm; INFORS AG, Bottmingen, Switzerland). For *H. pseudoflava* cultivations, a seed train from Grenz et al. (2019) was slightly modified. Cells from a 10% (v v^−1^) glycerol stock (stored at −80°C) were streaked on a TB agar plate and incubated for 3 days. Subsequently, a single colony was used to inoculate a test tube containing 5 mL LBo medium and incubated for approximately 8 h. The complete preculture was transferred into 50 mL CO‐Ox minimal medium with 10 g fructose L^−1^ and cultivated overnight (14–16 h). Unless stated otherwise, the main cultures were grown in 50 mL CO‐Ox minimal medium and inoculated to an optical density at 600 nm (OD_600_) of 0.25 (heterotrophic) or 0.1 (chemolithoautotrophic, mixotrophic). Heterotrophic cultivations were carried out in 500 mL baffled shaking flasks. In contrast, chemolithoautotrophic and mixotrophic cultivations were conducted in 500 mL serum bottles (total volume = 561.77 ± 7.56 mL) and sealed with a gastight lid. The headspace of a serum bottle was evacuated to < 40 mbar and thereafter filled with the respective gas mixture to an overpressure of 1 bar. This gasification step was repeated once to ensure a complete renewal of the gas atmosphere. The gas atmosphere was exchanged at specific time points throughout the gas fermentation, as illustrated in the figures (Siebert et al. 2020).

The OD_600_ was measured using an Ultrospec 10 cell density meter (Biochrom Ltd., Cambridge, UK) to monitor growth. The growth rate (*μ* | h^−1^) was determined graphically by applying exponential regression to the semi‐logarithmic plot of OD_600_ against time, and the coefficient of determination (R^2^) was maximized using at least four data points in the exponential phase.

### 2.3 Recombinant DNA Work

All oligonucleotides used are listed in Table S2 and were purchased from Sigma‐Aldrich Chemie GmbH (Taufkirchen, Germany). All enzymes were acquired from New England BioLabs GmbH (Frankfurt am Main, Germany) and used according to the manufacturer's protocol. DNA fragments were amplified with Q5 High‐Fidelity DNA Polymerase. Genomic DNA, PCR products and plasmids were purified using commercial kits (NucleoSpin Microbial DNA, NucleoSpin Gel and PCR Clean‐up, NucleoSpin Plasmid) from MACHEREY‐NAGEL GmbH & Co. KG (Düren, Germany), following the supplier's instructions. For genomic deletions by homologous recombination (double crossover), the plasmid pK19*mobsacB* (Schäfer et al. 1994) was linearized with SmaI, and homologous regions upstream and downstream of the target gene were inserted using Gibson assembly (Gibson et al. 2009). To express SH in *H. pseudoflava* , the structural genes and the gene for the endopeptidase HoxW were cloned into the BamHI linearized expression vector pOCEx1 (Grenz et al. 2019), with HoxF linked to a *Strep*‐tag II (Schmidt and Skerra 2007), as recently published in Siebert et al. (2026). The corresponding oligonucleotide included an artificial ribosomal binding site and spacer sequence (5′‐GAGGAGAACGGA‐3′) of the carboxydotrophic bacterium *A. carboxidovorans* (Siebert et al. 2020).

Chemically competent *E. coli* S17‐1 was transformed via heat shock at 42°C (Green and Sambrook 2012). Positive clones were verified by colony PCR using Quick‐Load *Taq* 2X Master Mix, and plasmid assemblies were validated via Sanger or Oxford Nanopore Sequencing (Microsynth Seqlab GmbH, Göttingen, Germany). *H. pseudoflava* was transformed by conjugation (Grenz et al. 2019), and colony PCR was used to confirm chromosomal deletions.

### 2.4 Proteomics

Five biological replicates of *H. pseudoflava* wild type (WT) were aerobically cultivated under four different conditions with a start‐OD_600_ of 0.1: (I) 10 g fructose L^−1^, (II) 10 g fructose L^−1^ under a gas atmosphere of 10% CO_2_, 86% H_2_ and 4% O_2_, (III) solely the H_2_/CO_2_/O_2_ gas mixture or (IV) 80% CO and 20% synthetic air. Five millilitre cell culture was harvested in the exponential phase (OD_600_ = 0.39 ± 0.04; 13,000 × *g*, 4°C, 10 min) and stored at −80°C. Cell pellets were thawed and treated with 5% (v v^−1^) sodium dodecyl sulphate in 100 mM tris(hydroxymethyl)aminomethane (Tris). The required volume was calculated based on the protein content, assuming that proteins accounted for approximately 50% of the CDW, with CDW derived from OD_600_ using the correlation factor of 0.277 g CDW L^−1^ OD^−1^ (Grenz et al. 2019). Fifty microlitre of a sample (approximately 50 μg protein) was supplemented with 4 μL 500 mM dithiothreitol and reduced for 1 h at 70°C, followed by alkylation with 8 μL 500 mM 2‐iodoacetamide for 30 min in the dark at room temperature. For acidification, 2.5 μL 27.5% (v v^−1^) phosphoric acid was added, and proteins were subsequently bound using 165 μL 90% (v v^−1^) methanol in 100 mM Tris. The liquid was transferred onto S‐Trap micro spin columns (ProtiFi), and the proteins bound onto the column were washed three times with 150 μL 90% (v v^−1^) methanol in 100 mM Tris via centrifugation (2000 × *g*, 1 min). Proteins were digested with 1 μg trypsin in 50 μL 50 mM ammonium bicarbonate buffer overnight at 37°C. Peptides were eluted stepwise with 5%, 50% and 75% (v v^−1^) acetonitrile (ACN) in 0.1% (v v^−1^) formic acid (FA) (40 μL of each eluent). Eluted peptides were dried in a vacuum centrifuge (Eppendorf Concentrator plus; Eppendorf SE, Hamburg, Germany) and resuspended in 20 μL 5% (v v^−1^) ACN in 0.1% (v v^−1^) FA.

For liquid chromatography–tandem mass spectrometry (LC–MS/MS) analysis, 2 μL of sample was injected into the Ultimate 3000 RSLCnano HPLC system (Thermo Fisher Scientific Inc., Waltham, MA, USA) with a Thermo Fisher Accaim PepMap C18 reverse phase trap column (300 μm × 5 mm × 5 μm). Ultrapure water and 80% (v v^−1^) ACN, both in 0.1% (v v^−1^) FA, were used as mobile phases at a flow rate of 20 μL min^−1^ to desalt the sample, which was then directed onto the Waters nanoEase^TM^ M/Z Peptide CSH C18 column (130 Å × 1.7 μm × 150 μm × 100 mm; Waters Corporation, Milford, MA, USA). Peptides were eluted using a gradient from 8% to 95% of 80% (v v^−1^) ACN in 0.1% (v v^−1^) FA for 60 min with a flow rate of 0.5 μL min^−1^. After separation with liquid chromatography, peptides were electron‐sprayed into the Orbitrap Eclipse Tribrid tandem MS system (Thermo Fisher Scientific Inc., Waltham, MA, USA). The MS/MS was performed at a resolution of 30,000, with a normalized collision energy of 30% in quadrupole isolation mode including three sections as follows: (I) *m/z* range 350–470, with isolation windows of 10 *m/z* with a 1 *m/z* overlap, (II) *m/z* range 465–645, with isolation windows of 5 *m/z* and a 1 *m/z* overlap, (III) *m/z* range 640–1100, with an isolation window of 46 *m/z* and a 1 *m/z* overlap.

Data‐independent acquisition data processing was performed using Spectronaut (version 19.0.240606.62635) (Bruderer et al. 2015) with default settings, and the collected mass spectra were searched against the corresponding databases (CP037867 [chromosome] and CP037868 [megaplasmid pDSM1084]) (Grenz et al. 2019). Proteins were identified as differentially abundant using the following cut‐off threshold (Heffernan et al. 2024): 1% false discovery rate, number of unique peptides ≥ 2, *p*‐value ≤ 0.05, *q*‐value ≤ 0.01 and fold change (FC) ≤ 0.667 and ≥ 1.5 (|log_2_FC| ≥ 0.585). The mass spectrometry proteomic data have been deposited to the ProteomeXchange Consortium via the PRIDE (Perez‐Riverol et al. 2025) partner repository, with the dataset identifier PXD068401.

### 2.5 Cell Extract Preparation

To determine the hydrogenase or CO dehydrogenase activity of *H. pseudoflava* cultivations, 40 mL cell culture was harvested in the exponential phase (4000 × *g*, 4°C, 10 min), washed once with a 50 mM potassium phosphate buffer (pH 7), and stored at −80°C. Thawed cell pellets were resuspended in 1 mL buffer and disrupted by sonication (Sonopuls HD 4050 Homogenizer; Bandelin Electronic GmbH & Co. KG, Berlin, Germany) with the following settings: amplitude, 100%; pulse, 0.5 s on/0.5 s off; duration, 1.5 min; one repeat; 5 min break on ice between runs. After the disruption, the cells were centrifuged (5000 × *g*, 4°C, 20 min). The supernatant (crude extract) was used to perform enzyme assays (Meyer and Schlegel 1979).

To analyse the in vivo activity of the SH, *H. pseudoflava* (pOCEx1‐*Strep*II‐*hoxFUYHW*) was cultivated in 200 mL CO‐Ox minimal medium with 10 g fructose L^−1^ in 2 L baffled shaking flasks. Cells were harvested during the exponential phase at an OD_600_ ≥ 4 (7800 × *g*, 4°C, 20 min), washed once with a 50 mM Tris–HCl, 150 mM KCl buffer (pH 7.5), resuspended in the same buffer (3 mL g^−1^ wet cell weight), and stored at −80°C. Cell suspension was lysed by sonication (UIS250V; Hielscher Ultrasonics GmbH, Teltow, Germany) with the following settings: amplitude, 60%; pulse, 0.5 s on/0.5 s off; duration, 3 min; two repeats; 1 min break on ice between runs and centrifuged for 1 h at 45,000 × *g* and 4°C to remove remaining non‐lysed cells, cell debris, and membrane fractions (cell‐free extract) (Siebert et al. 2026).

### 2.6 Enzyme Assays

CO and H_2_ oxidation were measured spectrophotometrically at 615 nm and 30°C in a 3 mL cuvette (Ø = 1 cm; Hellma GmbH & Co. KG, Müllheim, Germany), gastight closed with a rubber septum. The reaction mix (total volume of 2 mL) contained 50 mM gas‐saturated potassium phosphate buffer (pH 7) and 6 μL 50 mM methylene blue (*ε*_615_ = 37.1 mM^−1^ cm^−1^) as an artificial electron acceptor. The reaction was initiated by adding 25–200 μL of crude extract using a gastight Hamilton syringe (Hamilton Bonaduz AG, Bonaduz, Switzerland) (Meyer and Schlegel 1979; Lenz et al. 2018). The H_2_‐driven NAD^+^ reduction by SH was measured in cuvettes containing 50 mM H_2_‐saturated Tris–HCl, 1 mM NAD^+^, 1 mM tris(2‐carboxyethyl)phosphine and 1 μM FMN (pH 7). The reaction was started by the addition of 200 μL of cell‐free extract. Measurements were conducted at 365 nm (*ε*_365 nm_ = 3.3 mM^−1^ cm^−1^) (Schneider and Schlegel 1976; Siebert et al. 2026). Protein concentration was determined using the Bradford method (Bradford 1976).

## 3 Results

### 3.1 Chemolithoautotrophic Growth Drives Global Proteome Remodelling

To gain a comprehensive understanding of how *H. pseudoflava* adapts to mixotrophic and chemolithoautotrophic environments, we performed proteome analysis of cells grown under four conditions, harvested during the exponential growth phase (Figure 1A). Glucose is commonly used as a heterotrophic substrate in microbial biotechnology. In contrast to Grenz et al. (2019), we observed growth on glucose. However, it was slower than on fructose (*μ* = 0.29 ± 0.01 h^−1^; Figure 1A), and the fastest growth occurred after 48 h (*μ* = 0.16 ± 0.03 h^−1^; Figure S1A). Therefore, fructose was selected as glycolytic substrate for heterotrophic growth under ambient conditions (H) and for mixotrophic cultivation under a gas atmosphere of 10% CO_2_, 86% H_2_ and 4% O_2_ (M). The same gas composition was used to investigate chemolithoautotrophic growth with CO_2_ as sole carbon source and H_2_ as energy source (K; K referring to *Knallgas*‐like conditions). At last, CO (80% CO in synthetic air) served as both a carbon and an energy source (CO). For each condition, five independent cultivations (biological replicates) were performed. In total, 3863 proteins from the 4625 predicted coding sequences (Grenz et al. 2019) were quantified across all conditions; 3640 were identified based on at least two unique peptides (File S1). Principal component analysis (PCA) of the replicates revealed distinct clustering by growth condition (Figure S2). To evaluate the data, the protein abundances under chemolithoautotrophic or mixotrophic conditions were compared with those in the heterotrophic environment (log_2_FC for M/H, K/H and CO/H). The overall pattern of protein changes in the 3640 reliably quantified proteins across all three comparisons is visualized in volcano plots (Figure S3), and differentially abundant proteins relative to heterotrophic growth are summarized in a Venn diagram (Figure 1B). When comparing CO‐based growth to fructose‐based heterotrophic growth, 1003 proteins exhibited increased and 876 decreased abundance. In comparison, mixotrophic and CO_2_/H_2_ growth resulted in fewer proteins with altered abundance (in total 732 and 722, respectively). Accordingly, the CO condition is clearly separated from the other conditions in the PCA (Figure S2), highlighting the impact of CO on the proteome of *H. pseudoflava* .

**FIGURE 1:**
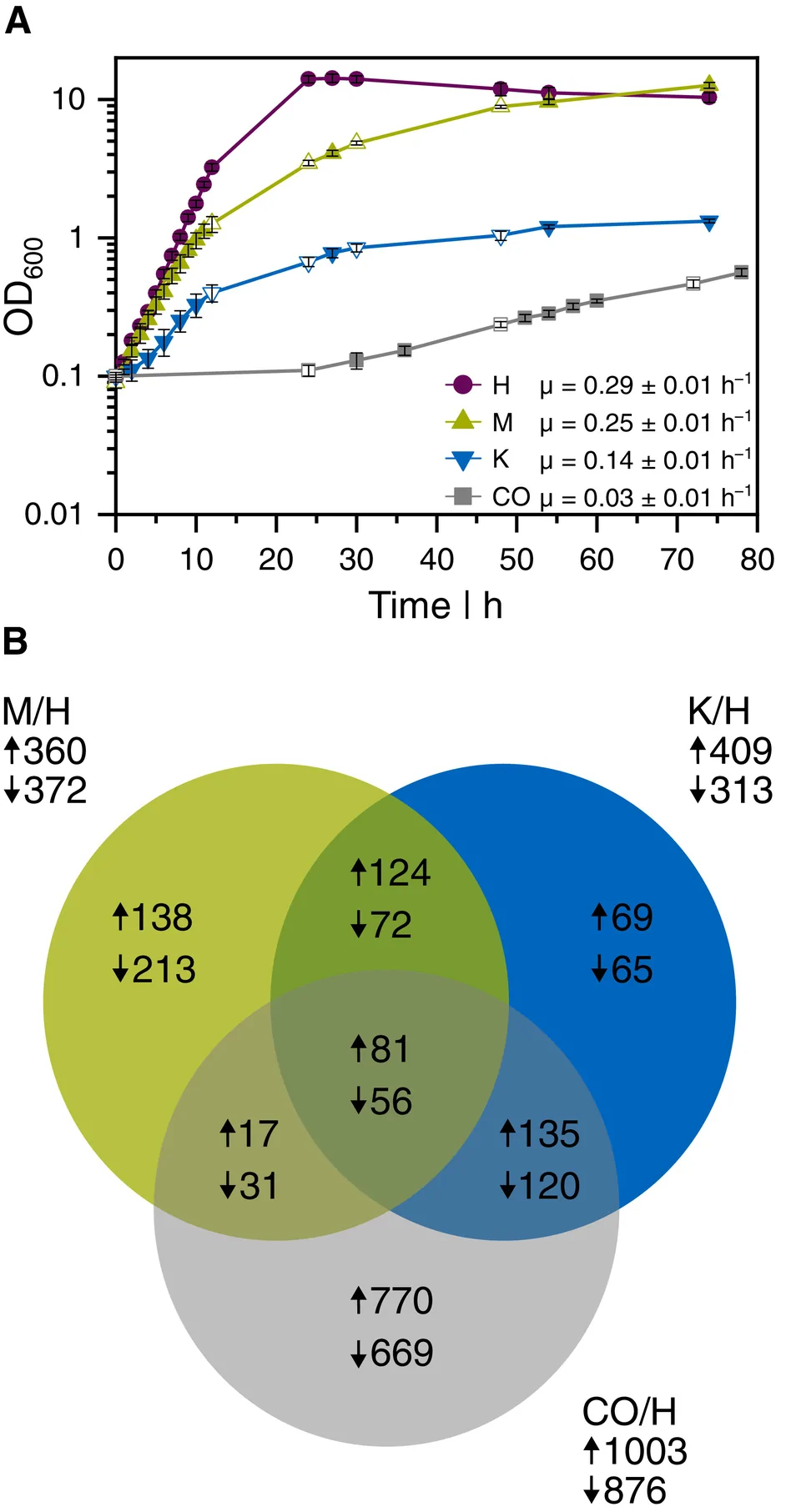
Growth (A) and proteomic changes (B) of *Hydrogenophaga pseudoflava* WT under heterotrophic (H), mixotrophic (M) and chemolithoautotrophic conditions (K and CO). H: 1% [w v^−1^] fructose (purple, circle); M: 1% [w v^−1^] fructose and a gas atmosphere of 10% CO_2_, 86% H_2_ and 4% O_2_ (green, triangle); K: Only the CO_2_/H_2_/O_2_ gas mixture (blue, inverted triangle); CO: Gas atmosphere of 80% CO and 20% synthetic air (grey, square). Cells were cultivated in 50 mL CO‐Ox minimal medium. (A) Open symbols denote time points for a complete headspace exchange. Data represents the mean, and error bars indicate the standard deviation of biological replicates (*n* ≥ 3). (B) VENN diagram (Heberle et al. 2015) of differentially abundant proteins comparing the cultivation conditions M, K and CO with H. More abundant proteins compared to the reference condition (H) are indicated with **↑**, and fewer abundant proteins with **↓**. Numbers displayed outside the VENN diagram indicate the total number of proteins with increased or decreased abundance for each comparison. Thresholds for comparisons: Unique number of peptides ≥ 2, *p*‐value ≤ 0.05, *q*‐value ≤ 0.01, FC ≤ 0.667 and ≥ 1.5 (|log_2_FC| ≥ 0.585). Proteome samples were obtained from biological replicates (*n* = 5).

### 3.2 Analysis of Proteins Related to Chemolithoautotrophic Metabolism

Across all three comparisons (M, K, CO vs. H), 81 proteins were more and 56 were less abundant (Figure 1B). Among the proteins with increased abundance were the enzymes of the CBB cycle, such as ribulose‐1,5‐bisphosphate carboxylase‐oxygenase (RuBisCO), which catalyses CO_2_ fixation (Figures 2 and 3). Deletion of the only annotated gene copy of the two subunits CbbS and CbbL resulted in the inability of *H. pseudoflava* to grow on the C1 substrates CO and CO_2_ (Figure S1B,C). Notably, Rossmann‐toolbox analysis (Kamiński et al. 2022), a computational approach for predicting cofactor specificity of Rossmann‐fold proteins, identified glyceraldehyde‐3‐phosphate dehydrogenase as NADH‐specific (GapA1: 99.28% probability, GapA2: 99.63%), supporting an NADH‐dependent CBB cycle in *H. pseudoflava* , similar to *C. necator* H16 (The UniProt Consortium 2025; Bansal et al. 2026). Most putative isoenzymes of the CBB cycle, such as GapA2, remained unchanged across all conditions (Table S3). Relative to heterotrophic growth, the abundance of 6‐phosphogluconate dehydratase and 2‐keto‐3‐deoxy‐6‐phosphogluconate aldolase, key enzymes of the Entner‐Doudoroff (ED) pathway, was strongly reduced under chemolithoautotrophic conditions (log_2_FC ≤ −1.30) (Table S3). Some proteins associated with the tricarboxylic acid cycle were also affected by the cultivation conditions (Table S3). Additionally, the enzymes of the glyoxylate shunt displayed changes in abundance under all three conditions compared to fructose‐grown cells. Isocitrate lyase showed higher presence under CO and CO_2_/H_2_, whereas malate synthase was increased under mixotrophic and CO_2_/H_2_ conditions (Table S3). The glycine cleavage system (GcvTHP) protein abundance also increased, except for GcvP under *Knallgas*. Additionally, the l‐serine dehydratase (SdaA) was more abundant in all comparisons (Table S3). GcvTHP and SdaA link glycine/serine catabolism to central carbon metabolism by generating single‐carbon units and pyruvate, respectively (Sarwar et al. 2016).

**FIGURE 2:**
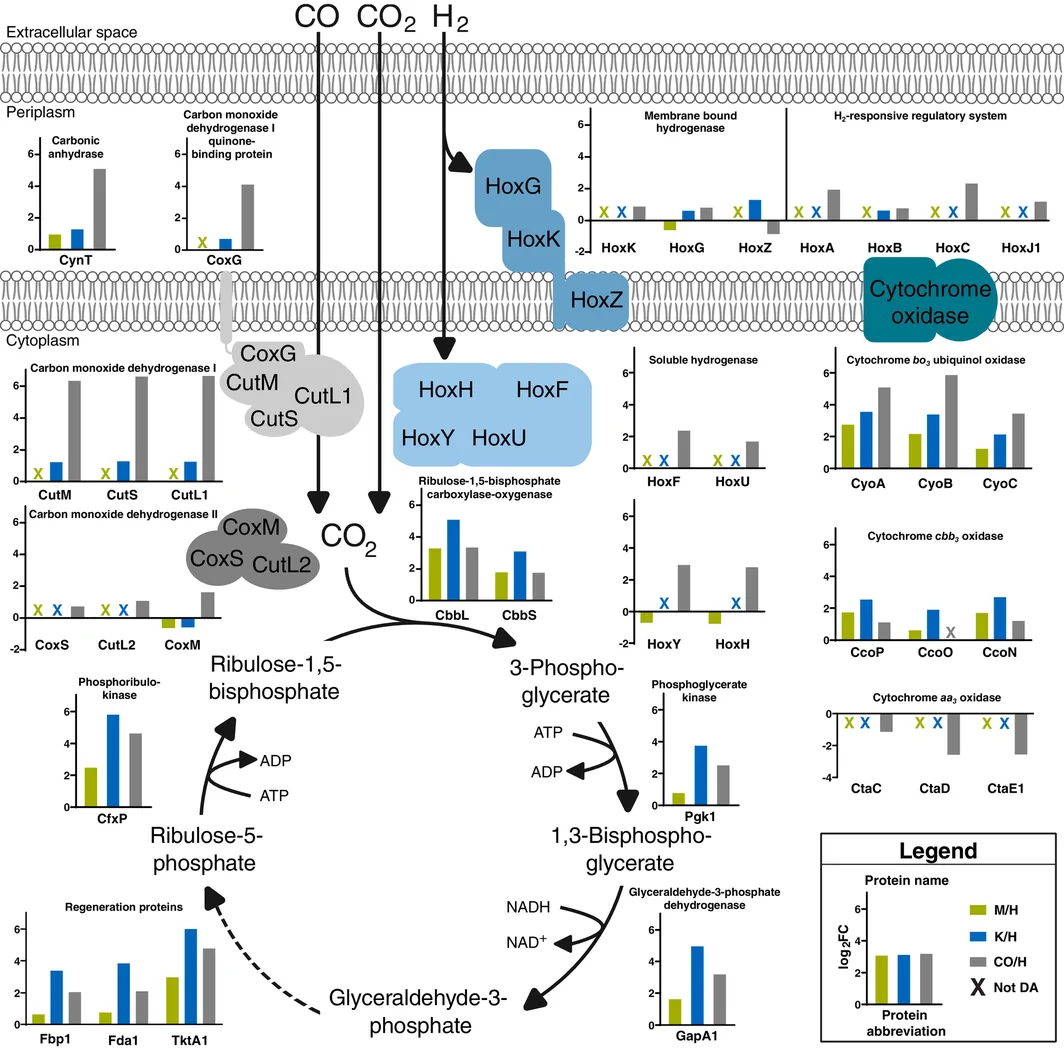
Changes in protein abundance within the core chemolithoautotrophic metabolism of *Hydrogenophaga pseudoflava* WT grown under mixotrophic (M, green), and chemolithoautotrophic conditions (K, blue and CO, grey) relative to heterotrophic growth (H). Bar graphs display log_2_FC of protein abundance. Proteins were defined as differentially abundant (DA) based on the following thresholds: Unique peptides ≥ 2, *p*‐value ≤ 0.05, *q*‐value ≤ 0.01 and FC ≤ 0.667 or ≥ 1.5 (|log_2_FC| ≥ 0.585). A X indicates no differential abundance. Proteomic samples were obtained from biological replicates (*n* = 5). CbbLS, large/small subunit of RuBisCO; CcoPON, cytochrome *cbb*_*3*_ oxidase subunits; CfxP, phosphoribulokinase; CoxG, CODH I subunit G; CoxSL2M, small/large/medium subunit of CODH II; CtaCDE1, cytochrome *aa*_*3*_ oxidase subunits; CutMSL1, medium/small/large subunit of the CODH I; CynT, carbonic anhydrase; CyoABC, cytochrome *bo*_*3*_ ubiquinol oxidase subunits; Fbp1, fructose‐1,6‐bisphosphatase; Fda1, fructose‐bisphosphate aldolase; GapA1, glyceraldehyde‐3‐phosphate dehydrogenase; HoxA, DNA‐interacting response regulator; HoxBC, small/large subunit of the cytoplasmic regulatory [NiFe]‐hydrogenase; HoxFUYH, alpha/gamma/delta/beta subunit of SH; HoxJ1, subunit of histidine protein kinase; HoxKG, small/large subunit of MBH; HoxZ, [NiFe]‐hydrogenase *b*‐type cytochrome; Pgk1, phosphoglycerate kinase; TktA1, transketolase. Additional details on protein annotation and proteomics data are provided in Table S3.

**FIGURE 3:**
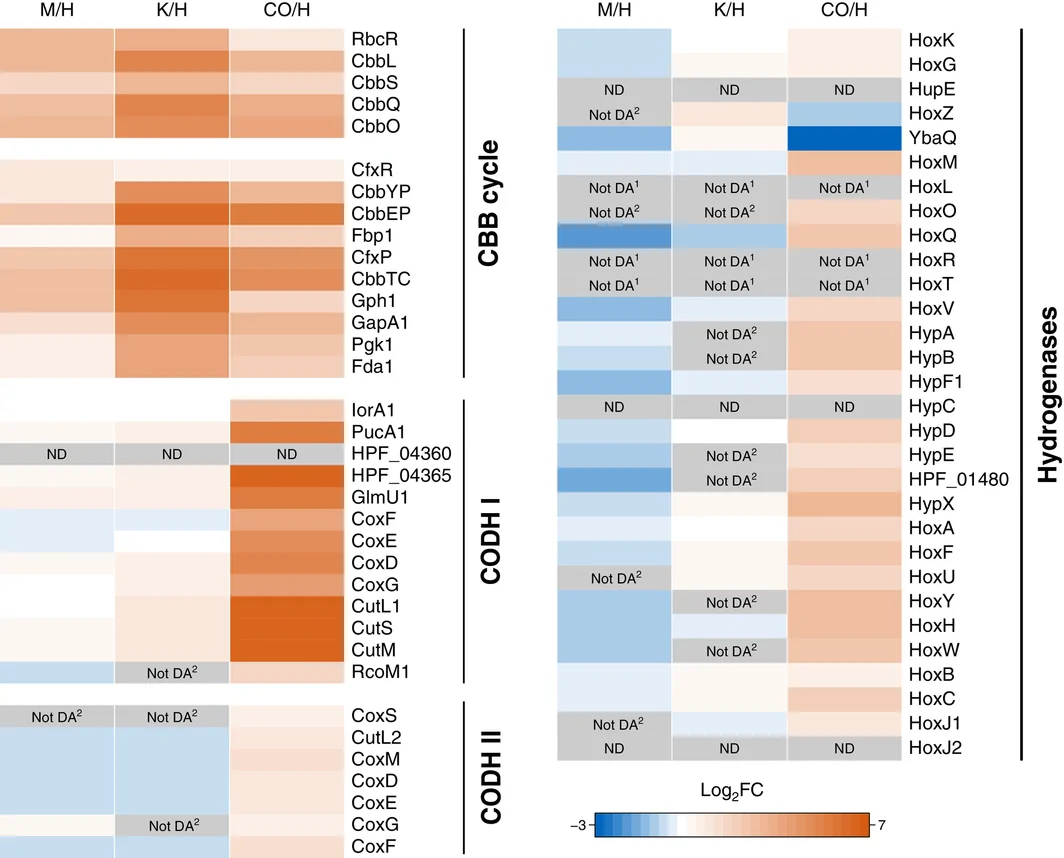
Global overview of protein changes (log_2_FC) related to CODHs, hydrogenases, and the CBB cycle in *Hydrogenophaga pseudoflava* WT grown mixotrophically and chemolithoautotrophically relative to heterotrophic growth (M, K, CO vs. H). Proteome samples were obtained from biological replicates (*n* = 5). ND, not detected; Not DA, not differentially abundant based on defined cut‐off threshold: DA^1^, unique peptides not ≥ 2; DA^2^, unique peptides ≥ 2, but not *p*‐value ≤ 0.05, *q*‐value ≤ 0.01. Additional details on protein annotation and proteomics data are provided in Table S3.

Interestingly, the proteins encoded by the hydrogenase cluster did not show increased abundance under a CO_2_/H_2_ atmosphere compared with heterotrophic growth on fructose (Figure 3). Only HoxZ reached a log_2_FC > 1 under *Knallgas* condition. In *C. necator* H16, HoxZ anchors the MBH to the membrane and transfers electrons from HoxKG to the quinone pool (Bernhard et al. 1997). The other hydrogenase subunits remained largely unchanged (Figure 2). In contrast, most proteins within this cluster exhibited higher abundances under CO (log_2_FC ≥ 0.77), including the structural genes of the SH (log_2_FC ≥ 1.68) (Figure 2), but excluding HoxZ and the transcriptional regulator YbaQ (Figure 3). Under the mixotrophic condition, the cluster displayed predominantly negative log_2_FC (Figure 3). The two CODH clusters showed clear differences in abundance. The CODH I cluster was strongly increased (log_2_FC ≥ 1.78) under CO growth (Figure 3). In particular, the three subunits CutMSL1 displayed high log_2_FC values (≥ 6.35), among the highest observed (Figure 2). Under CO_2_/H_2_ atmosphere, most CODH I proteins were more abundant, whereas under the mixotrophic condition, most remained unchanged (Figure 3). In contrast to CODH I, the Form II cluster showed a lower increase under CO (0.73 ≤ log_2_FC ≤ 1.65) and was only slightly affected under the other conditions (Figure 3).

Among the putative carbonic anhydrases (Cah, Can1, Can2, CynT), CynT appears to be the primary enzyme supplying CO_2_ to RuBisCO (Figure 2), as indicated by pronounced log_2_FC, particularly high under CO condition (log_2_FC = 5.08), whereas the other annotated variants remained unchanged (Cah was not detected) (Table S3). Paul et al. (2011) observed that adaptation of *A. carboxidovorans* OM5 to chemolithoautotrophic growth involved changes in proteins related to oxidative homeostasis, suggesting that redox balance is an important aspect of cellular adaptation. Riboflavin is required for the central redox cofactors FMN and FAD. Proteins within the riboflavin synthesis also differed between our conditions: RibBA1 and RibD were highest in CO‐grown cells, whereas RibBA2 predominated under CO_2_/H_2_ conditions (M, K). RibBA1 level was also higher under *Knallgas* than under fructose (Table S3). Furthermore, the conserved two‐component redox‐sensing system RegAB was more abundant during chemolithoautotrophic growth (log_2_FC ≥ 0.64), with RegA also showing higher levels under mixotrophy (log_2_FC ≥ 1.39) (Table S3). Under reduced oxygen concentrations present in M, K and CO compared to H (4% vs. 21%), denitrification represents an alternative respiratory pathway, with denitrification‐related proteins exhibiting distinct abundance patterns: nitrate reductase was more abundant under CO, whereas nitric oxide, nitrite and nitrous oxide reductases were higher under a CO_2_/H_2_ atmosphere (M, K) (Table S3). However, earlier work demonstrated that *H. pseudoflava* cannot grow on CO or CO_2_/H_2_ when nitrate or nitrite is provided as the terminal electron acceptor, whereas growth was observed with organic substrates (Frunzke and Meyer 1990).

A key trait of carboxydotrophic bacteria is their branched respiratory chain, which consists of a heterotrophic and an autotrophic branch, each with distinct cytochromes and terminal oxidases (Cypionka and Meyer 1983a; Siebert et al. 2021). The annotated cytochrome *aa*_*3*_ oxidase (CtaCDE), typically utilized at high O_2_ concentrations, showed markedly lower abundance (log_2_FC ≤ −1.14) when CO was used as the carbon and energy source (Figure 2). Assembly factors CtaB and CtaG also decreased under H_2_‐based growth, and CtaG even under mixotrophic condition (Table S3). In contrast, cytochrome *bo*_*3*_ ubiquinol oxidase (CyoABCD) was more abundant under all three conditions relative to heterotrophic growth (log_2_FC ≥ 1.23; CyoD was not detected), with the strongest increase under CO, followed by CO_2_/H_2_ and mixotrophic condition (Figure 2). Cytochrome *cbb*_*3*_ oxidase (CcoPQON) was most abundant when using H_2_ as the sole energy source (log_2_FC ≥ 1.89; CcoQ was not detected) (Figure 2). The *bc*_*1*_ cytochrome reductase (PetABC) did not change in any comparison (Table S3). Furthermore, several putative cytochromes displayed condition‐specific abundance. Some were more frequently detected under CO_2_/H_2_, particularly in mixotrophic growth, but less so under CO (*c*‐type: HPF_07360 [C2], HPF_17355 [C‐555]). Similarly, an annotated cytochrome *c* (HPF_22955), with predicted peroxidase‐related activity (Blum et al. 2025), showed lower abundance under CO (Table S3). Other cytochromes were significantly more frequent under CO than under the other three conditions (*b*‐type: HPF_02100 [HyaC]; *c*‐type: HPF_02105 [C′], HPF_21835 [Cc2]). Additionally, three further annotated cytochromes had markedly increased protein levels under the tested CO condition: an alcohol dehydrogenase cytochrome (AdhB), a biotin biosynthesis‐related cytochrome (BioI), and an annotated cytochrome P450 (HPF_14080). The latter is predicted to function as a monooxygenase or peroxidase (Blum et al. 2025) (Table S3).

Moreover, growth on CO was associated with a stress response. The abundance of universal stress proteins was altered, and the protein levels of various regulatory components were increased (Table S3). This involved serine/threonine protein kinases (e.g., PrkC, PknB) and the corresponding phosphatase Stp2, as well as extracytoplasmic function (ECF) sigma factors SigG, SigL and SigR1 (log_2_FC ≥ 0.95). In addition, proteins associated with cell envelope homeostasis were differentially abundant, including enzymes involved in peptidoglycan remodelling (e.g., CwlK, PgdA homologues), periplasmic protein quality control (Chaperone SurA1, DegP‐, AprX‐, CtpA‐ and subtilisin‐like proteases), Ugp transport system, and disulphide bond formation protein DsbB (Table S3). Other proteins suggest an oxidative stress response in CO‐grown cells (Table S3). The catalase‐peroxidase KatG showed increased abundance under both chemolithoautotrophic conditions, particularly under CO (log_2_FC = 2.19). Additionally, an annotated heme peroxidase (HPF_20145) was strongly enriched (log_2_FC = 4.52). The two putative NADPH dehydrogenases, NamA1 and NamA2, changed in protein abundance (log_2_FC = −0.69 and 1.12, respectively). Other notable features include increased presence of the NuoF2 subunit of the NADH dehydrogenase complex (log_2_FC = 0.98) and of proteins involved in pyrroloquinoline quinone (PQQ) biosynthesis (log_2_FC ≥ 1.13) (Table S3). No *pqqA* gene could be identified in the genome, consistent with its frequent absence in bacterial genome annotations (Shen et al. 2012). Furthermore, various dehydrogenases were altered under CO, including higher abundances of quinoprotein ethanol dehydrogenase (QedA), quinoprotein amine dehydrogenase (QhnDH) and l‐lactate dehydrogenase [cytochrome] (LldD) (log_2_FC ≥ 1.67) (Table S3).

### 3.3 Relevance of Hydrogenases Under CO_2_ /H_2_ Growth Conditions

As a hydrogen‐oxidizing bacterium, *H. pseudoflava* can use hydrogen as an alternative energy source alongside organic substrates or CO. The MBH, encoded by the structural genes *hoxKG* and presumably anchored via the membrane protein HoxZ, has been described previously (Cypionka et al. 1980; Grenz et al. 2019; Siebert et al. 2021). Furthermore, the corresponding gene cluster encodes key maturation factors and a putative SH (*hoxFUYH*) (Grenz et al. 2019; Siebert et al. 2021). Deletion mutants were generated to investigate the functional relevance of both hydrogenases for growth and overall hydrogenase activity. Cells were cultivated chemolithoautotrophically with a CO_2_/H_2_/O_2_ gas mixture or heterotrophically with fructose. *H. pseudoflava* Δ*hyd*, which lacks the entire hydrogenase cluster, did not grow under this chemolithoautotrophic condition (Figure S1B). Likewise, deletion of *hoxKG* caused a complete growth defect, demonstrating that the MBH is essential for H_2_‐dependent energy generation (Figure 4A). Furthermore, cell extracts of the Δ*hoxKG* mutant showed almost no detectable hydrogenase activity using methylene blue as an artificial electron acceptor (1 ± 0 nmol H_2_ min^−1^ mg^−1^ protein) (Figure 4B). In contrast, the Δ*hoxFUYH* mutant showed similar H_2_‐oxidizing activity and growth behaviour as the WT, with a comparable growth rate of 0.15 ± 0.01 h^−1^ (WT: 0.14 ± 0.01 h^−1^), suggesting that the SH is inactive under the tested conditions (Figure 4A,B). Because HoxFUYH subunits showed increased protein abundance under CO relative to other conditions (Figure 2), the mutant's growth behaviour was also evaluated on 80% CO in synthetic air, with no differences in growth being observed (Figure S1D).

**FIGURE 4:**
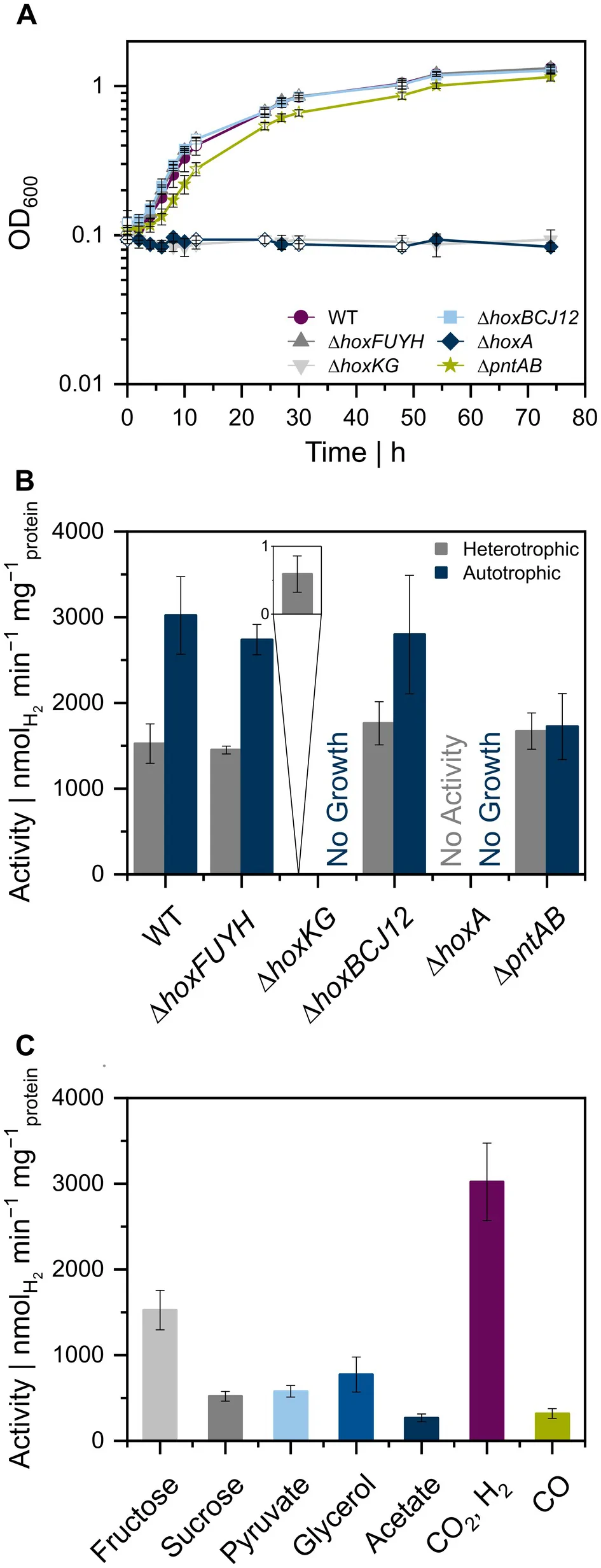
Characterization of *Hydrogenophaga pseudoflava* WT and mutant strains lacking key genes for putative hydrogenases or the transhydrogenase PntAB. (A) Chemolithoautotrophic growth of WT (purple, circle), Δ*hoxFUYH* (dark grey, triangle), Δ*hoxKG* (light grey, inverted triangle), Δ*hoxBCJ12* (light blue, square), Δ*hoxA* (dark blue, diamond) and Δ*pntAB* (green, star) in 50 mL CO‐Ox minimal medium under a gas atmosphere containing 10% CO_2_, 86% H_2_ and 4% O_2_. Open symbols denote time points for a complete headspace exchange. (B) Specific hydrogenase activity in crude extracts of WT, Δ*hoxFUYH*, Δ*hoxKG*, Δ*hoxBCJ12*, Δ*hoxA* and Δ*pntAB* cultivated either heterotrophically with 1% [w v^−1^] fructose (grey) or chemolithoautotrophically with the CO_2_/H_2_/O_2_ gas mixture (blue). (C) Specific hydrogenase activity in crude extracts of the WT grown on different carbon and energy sources: 1% [w v^−1^] fructose, 1% [w v^−1^] sucrose, 1% [w v^−1^] pyruvate, 1% [w v^−1^] glycerol, 0.1% [w v^−1^] acetate, CO_2_/H_2_/O_2_ gas mixture or CO gas containing 80% CO and 20% synthetic air. Data represents the mean, and error bars indicate the standard deviation of biological replicates (*n* ≥ 3).

Recently, Siebert et al. (2026) characterized the SH in vitro. To investigate the in vivo function of this enzyme, total hydrogenase activity was measured in *H. pseudoflava* (pOCEx1‐*Strep*II‐*hoxFUYHW*) (Siebert et al. 2026) grown on 1% [w v^−1^] fructose. Since electron transfer from H_2_ to NAD^+^ occurred (321 ± 20 nmol H_2_ min^−1^ mg^−1^ protein), the SH was confirmed to be active in the cell‐free extract (Figure S4A). These findings led to further investigations of whether the SH of *H. pseudoflava* supports energy generation under aerobic CO_2_/H_2_ conditions and can compensate for MBH under hydrogen‐rich conditions. Despite overexpression of the structural genes and the endopeptidase, *H. pseudoflava* Δ*hoxKG* (pOCEx1‐*Strep*II‐*hoxFUYHW*) failed to grow solely on CO_2_/H_2_/O_2_ (Figure S4B), indicating that the SH alone is insufficient to enable growth under the applied condition.

Furthermore, genome analysis indicated the presence of a potential H_2_‐sensing regulatory system (*hoxABCJ12*) homologous to that of *C. necator* H16 (Siebert et al. 2021). Inactivation of the response regulator HoxA in *H. pseudoflava* resulted in a complete loss of growth when hydrogen was provided as the sole electron donor (Figure 4A). Additionally, cell extracts from the Δ*hoxA* mutant failed to reduce methylene blue with H_2_ as the substrate (Figure 4B). In contrast, the Δ*hoxBCJ12* mutant, lacking genes encoding the putative regulatory hydrogenase HoxBC and the histidine kinase HoxJ, exhibited growth and H_2_‐oxidizing activity comparable to the WT (Figure 4A,B). In the genome of *H. pseudoflava* , the stop codon of *hoxJ1* overlaps with the start codon of *hoxJ2*, suggesting that a point mutation may have split the original *hoxJ* gene into two artificial subunits. Notably, HoxJ2 was not detected in any proteome under any tested condition (Table S3).

Highest hydrogenase activity using methylene blue was observed under CO_2_/H_2_ (3021 ± 452 nmol H_2_ min^−1^ mg^−1^ protein) (Figure 4C). We found that hydrogenase activity was also present under heterotrophic cultivations in a substrate‐dependent manner. Besides the similar abundance of the hydrogenase‐related proteins under fructose compared to CO_2_/H_2_ (Figure 3), a notable activity of 1525 ± 230 nmol H_2_ min^−1^ mg^−1^ protein was measured in cells grown on fructose (Figure 4C). Other organic substrates led to hydrogenase activity between 267 and 774 nmol H_2_ min^−1^ mg^−1^ protein (Figure 4C). Although the proteins of the hydrogenase cluster showed significant abundance under CO (Figure 3), the overall activity was rather low at 319 ± 57 nmol H_2_ min^−1^ mg^−1^ protein. Interestingly, hydrogenase activity correlated with HoxZ abundance, with the highest levels under CO_2_/H_2_, followed by fructose, and the lowest under CO, indicating the relevance of HoxZ and the whole MBH for hydrogenase activity (Figure 4C).

### 3.4 Impact of the Transhydrogenase PntAB on Chemolithoautotrophic Growth

The membrane‐bound transhydrogenase PntAB plays an important role in maintaining cellular redox balance in many organisms (Zhang et al. 2017). It catalyses the hydride transfer between NADH and NADP^+^, a process coupled to proton translocation across the membrane (Jackson 2003). The genome of *H. pseudoflava* also encodes PntAB (Grenz et al. 2019). To evaluate its relevance for the chemolithoautotrophic metabolism, we deleted the genes and conducted growth experiments under various conditions. Although no differences in growth between the WT and the Δ*pntAB* mutant were observed on fructose (Figure S5A), the mutant displayed a pronounced lag phase (approximately 4 h) under chemolithoautotrophic growth with CO_2_ and H_2_. In addition, it exhibited a slightly reduced growth rate (0.12 ± 0.01 vs. 0.14 ± 0.01 h^−1^) and final OD_600_ (1.15 ± 0.07 vs. 1.32 ± 0.05) compared to the WT (Figure 4A). A comparable pattern was observed under CO, despite an unchanged growth rate (Figure S5B). To further investigate the role of PntAB, MgSO_4_, CaCl_2_, ferric ammonium citrate, and the trace element solution SL‐6 were added at one‐tenth of their standard concentrations. Reducing these supplements led to a distinct increase in the lag phase of the Δ*pntAB* mutant, whereas the WT adapted faster (around 10 and 2 h, respectively) (Figure S5C). Further experiments revealed that reduced availability of ferric ammonium citrate primarily accounts for the observed phenotype (Figure S5D). In addition to the observed growth effects, deletion of the *pntAB* genes affected hydrogenase activity during chemolithoautotrophic growth with H_2_ as the energy source and unreduced supplements. Using methylene blue, the overall activity in the crude extract of the Δ*pntAB* strain decreased to 1725 ± 386 nmol H_2_ min^−1^ mg^−1^ protein (WT: 3021 ± 452 nmol H_2_ min^−1^ mg^−1^ protein), reaching levels similar to those observed during heterotrophic cultivation with fructose (1672 ± 212 nmol H_2_ min^−1^ mg^−1^ protein) (Figure 4B). These results indicate a relevant role of the transhydrogenase PntAB during chemolithoautotrophic growth.

### 3.5 Functional Analysis of Carbon Monoxide Dehydrogenases During Growth on CO

The O_2_‐tolerant CODH enables carboxydotrophic bacteria to metabolize CO as both a carbon and energy source. Genome sequencing of *H. pseudoflava* revealed the presence of two putative CODH gene clusters, suggesting the coexistence of two distinct types of CODHs belonging to the OMP (CODH I) and BMS group (CODH II) (Grenz et al. 2019; Siebert et al. 2021). To determine which of these clusters are essential for growth under the tested high CO condition (80% CO, 20% synthetic air), deletion mutants lacking either the entire gene clusters (Δ*codh1* and Δ*codh2*) or the corresponding structural genes *cutMSL1* (CODH I) and *coxSL2M* (CODH II) were constructed. The Δ*cutMSL1* and Δ*codh1* mutants did not grow on 80% CO (Figures 5A and S1C, respectively). In contrast, the Δ*coxSL2M* strain displayed a growth phenotype similar to that of the WT. After a lag phase (the preculture contained fructose), cells entered exponential growth at a growth rate of 0.03 ± 0.01 h^−1^, reaching an OD_600_ of 1.27 ± 0.06 after 192 h and five headspace exchanges (Figure 5A). Even with the complete deletion of the CODH II cluster, *H. pseudoflava* Δ*codh2* maintained the ability to grow under this condition (Figure S1C). We also determined the CO‐oxidizing activity of cell extracts using methylene blue as an artificial electron acceptor. The WT strain cultivated chemolithoautotrophically with 80% CO exhibited a total activity of 1382 ± 56 nmol CO min^−1^ mg^−1^ protein (Figure 5B). By comparison, cultures grown heterotrophically with fructose showed only minimal activity (3 ± 1 nmol CO min^−1^ mg^−1^ protein). The Δ*coxSL2M* mutant behaved similarly to the WT, but the Δ*cutMSL1* strain exhibited no detectable activity heterotrophically (Figure 5B). Thus, CODH I is essential for growth on 80% CO.

**FIGURE 5:**
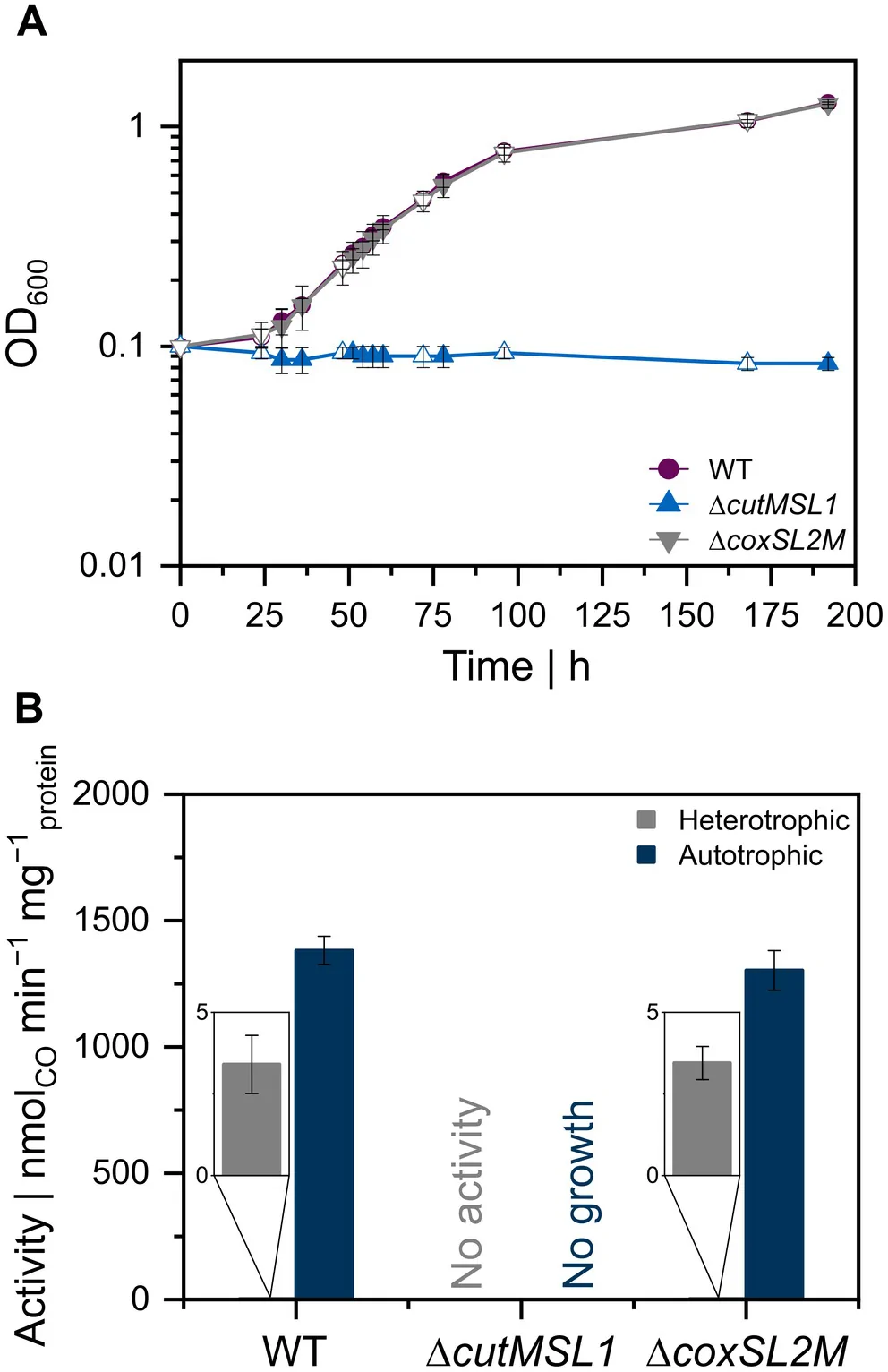
Growth (A) and specific CODH activity (B) of *Hydrogenophaga pseudoflava* WT, Δ*cutMSL1* and Δ*coxSL2M*. (A) WT (purple, circle), Δ*cutMSL1* (blue, triangle) and Δ*coxSL2M* (grey, inverted triangle) were grown in 50 mL CO‐Ox minimal medium under a gas atmosphere containing 80% CO and 20% synthetic air. Open symbols denote time points for a complete headspace exchange. (B) Specific CODH activity in crude extracts of WT, Δ*cutMSL1* and Δ*coxSL2M* cultivated either heterotrophically with 1% [w v^−1^] fructose (grey) or chemolithoautotrophically with the CO gas mixture (blue). Data represents the mean, and error bars indicate the standard deviation of biological replicates (*n* ≥ 3).

## 4 Discussion

*Hydrogenophaga pseudoflava* DSM 1084 is a promising host for aerobic syngas fermentation, and additionally possesses relevant traits for mixotrophic fermentation concepts aimed to improve carbon efficiency during the utilization of heterotrophic carbon sources (Fast and Papoutsakis 2012; Grenz et al. 2019; Bertrand et al. 2026). Kiessling and Meyer (1982) demonstrated that H_2_ and CO can serve as additional energy sources alongside pyruvate, increasing biomass formation by 35% and 7%, and pyruvate incorporation by 27% and 12%, respectively, while reducing pyruvate oxidation. However, no additional CO_2_ was incorporated, and the key enzymes of the CBB cycle showed no activity on pyruvate in the presence of CO (Kiessling and Meyer 1982). Under the tested mixotrophic condition (fructose, CO_2_, H_2_), hydrogenase proteins were slightly less abundant than under fructose alone. In contrast, CBB cycle proteins showed significantly increased protein levels, with CbbL and CbbS showing log_2_FC values of 3.28 and 1.79, respectively (Figures 2 and 3). Also, the CbbR homologues RbcR, upstream of the RuBisCO genes (log_2_FC = 3.11), and CfxR, upstream of the CBB cycle operon (log_2_FC = 1.28), were more abundant, along with a moderate increase in RegA (log_2_FC = 1.39) under the mixotrophic condition (Figure 3, Table S3). In *C. necator* H16, the CBB cycle regulator CbbR interacts with RegA, the response regulator of the redox‐sensitive two‐component system RegAB (Elsen et al. 2004), linking CO_2_ fixation and redox homeostasis (Gruber et al. 2017). Nevertheless, increased CBB protein abundance alone does not demonstrate active CO_2_ fixation, as CBB proteins have also been observed during heterotrophic growth of *C. necator* H16 without an apparent fitness benefit (Jahn et al. 2021). Thermodynamic modelling of *C. necator* H16 predicted that H_2_ oxidation can support CO_2_ reassimilation and improve carbon efficiency, although forcing complete carbon conversion was thermodynamically less favourable than CO_2_ excretion for PHB production from fructose (Weldon and Euler 2025). Recent experimental work showed that *C. necator* H16 predominantly consumed fructose in medium with fructose, CO_2_, and H_2_, as hydrogenase expression was suppressed in the presence of fructose. However, simultaneous mixotrophic growth could be achieved by inducing the hydrogenase regulator HoxA to activate hydrogenase expression (Pu et al. 2026). *H. pseudoflava* retained measurable hydrogenase activity during heterotrophic growth on fructose (Figure 4C). Together with the increased abundance of CBB proteins and regulators under the mixotrophic condition, this may indicate a capacity for simultaneous utilization of fructose, CO_2_ and H_2_, although metabolic preparedness for a subsequent transition to chemolithoautotrophic growth cannot be excluded.

Hydrogenase expression in *H. pseudoflava* under H_2_‐free conditions is likely due to a disrupted H_2_‐sensing regulatory system (HoxBCJ12). In *C. necator* H16, H_2_ is sensed by the regulatory hydrogenase HoxBC, which interacts with the histidine kinase HoxJ. In the presence of H_2_, HoxJ autophosphorylation is inhibited, leading to dephosphorylation of the transcriptional regulator HoxA and activation of the hydrogenase genes (Lenz and Friedrich 1998; Buhrke et al. 2004). However, the H_2_‐sensing system in *C. necator* H16 is not functional due to a mutation in *hoxJ* (Lenz and Friedrich 1998). Similar to *C. necator* H16, deletion of *hoxA* abolished hydrogenase activity and prevented growth on CO_2_/H_2_, whereas *H. pseudoflava* Δ*hoxBCJ12* showed growth and hydrogenase activity comparable to the WT (Figure 4A,B) (Lenz and Friedrich 1998; Jahn et al. 2024). Additionally, HoxJ2 was not detected in the proteome (Table S3), suggesting that the C‐terminal fragment is either not produced or does not stably associate with HoxJ1. These observations suggest that the H_2_‐sensing regulatory system in *H. pseudoflava* is likely impaired, leading to constitutive expression of hydrogenase genes. Hydrogenase expression in *C. necator* H16 is also regulated by substrate quality via the σ^54^ sigma factor RpoN (Schwartz et al. 1998; Jahn et al. 2024). This layer of regulation also appears conserved in *H. pseudoflava* , as hydrogenase activity varied with the carbon source (Figure 4C). The original isolation of *H. pseudoflava* under elevated O_2_ concentration (20%) may have imposed selective pressure favouring variants that escaped O_2_‐sensitive regulation of hydrogenase expression while retaining carbon catabolite control (Zavarzin and Nozhevnikova 1977; Lenz et al. 2015).

In contrast to *C. necator* H16 (Schink and Schlegel 1978; Kömen et al. 1992; Jahn et al. 2024), aerobic H_2_‐dependent growth in *H. pseudoflava* is exclusively mediated by MBH, which is also the main hydrogenase in other carboxydotrophic bacteria (Bowien and Schlegel 1981). Inactivation of the SH did not alter the growth phenotype (Figure 4A), and overexpression of SH did not restore growth of the Δ*hoxKG* mutant lacking MBH activity (Figure S4B). However, a recent study confirmed the functionality of the purified SH from *H. pseudoflava* and demonstrated that the enzyme is strongly inhibited by oxygen in vitro (Siebert et al. 2026). The in vivo results of the present study support this hypothesis and indicate that SH may contribute to redox balancing under other conditions, such as low or absent oxygen, as described for hydrogenases in *C. necator* strains (Kuhn et al. 1984) and *M. smegmatis* mc^2^155 (Berney et al. 2014).

In heterotrophic organisms such as *Vibrio natriegens* ATCC 14048, the membrane‐bound transhydrogenase PntAB contributes substantially to cellular NADPH generation, thereby playing a key role in redox homeostasis (Long et al. 2017). Here, we found that inactivation of PntAB reduced overall hydrogenase activity during CO_2_/H_2_ growth, resulting in levels comparable to those under heterotrophic growth (Figure 4B). This adjustment may be mediated by the redox state sensing RegAB system, which has been shown to control hydrogenase gene expression in *Rhodopseudomonas palustris* CGA010 (Rey et al. 2006). Inactivation of PntAB could therefore alter the redox state and lead to repression of hydrogenase genes in *H. pseudoflava* . The pronounced lag phase observed under chemolithoautotrophic conditions (Figures 4A and S5B) underscores the importance of PntAB for redox control during chemolithoautotrophic growth. This growth delay was further prolonged by limited iron availability (Figure S5D), suggesting that PntAB‐mediated redox homeostasis may become particularly relevant for adaptation to conditions that challenge the cellular redox state, given iron's central role in electron transfer and regulatory processes (Beinert et al. 1997; Lill 2009). In contrast to autotrophic conditions, deletion of the *pntAB* genes did not affect growth on fructose (Figure S5A), potentially due to alternative routes for NADPH formation. Some bacteria use the ED pathway despite the presence of a complete Embden–Meyerhof–Parnas pathway, although the latter yields more ATP, with increased NADPH generation as one proposed advantage of ED pathway utilization (Klingner et al. 2015). In addition to the increased abundance of key ED pathway enzymes in *H. pseudoflava* , glucose‐6‐phosphate dehydrogenase and 6‐phosphogluconolactonase were also more abundant under both fructose‐containing conditions (Table S3). As the oxidative pentose phosphate pathway is incomplete in *H. pseudoflava* due to the absence of 6‐phosphogluconate dehydrogenase (Grenz et al. 2019), this further supports the preferential use of the ED pathway, thereby supplying NADPH and potentially compensating for the loss of PntAB.

Besides H_2_, aerobic carboxydotrophic bacteria can oxidize CO via an O_2_‐insensitive CODH, additionally generating CO_2_. In *H. pseudoflava* , two CODH forms are annotated: one belongs to the OMP group (Form I) and the other to the BMS group (Form II) (Grenz et al. 2019). Our data demonstrate that Form I CODH is essential for growth on CO and that Form II CODH cannot compensate for its loss (Figure 5A). In the literature, CODH activity has been reported in *H. pseudoflava* grown on pyruvate or glucose (Conrad et al. 1981; Kiessling and Meyer 1982). Substrate‐dependent effects, possibly linked to carbon catabolite repression, have been suggested, though the underlying mechanisms are largely unknown (Dent et al. 2023). For example, in *M. smegmatis* mc^2^155, CODH expression increases under carbon limitation, supporting survival via atmospheric CO as a supplemental energy source (Cordero et al. 2019). During growth on fructose, we detected only minor CODH activity (Figure 5B), indicating a tight regulation by the transcriptional regulator RcoM1 upstream of the CODH I cluster. However, proteomic data revealed increased abundance of CODH I proteins under CO_2_/H_2_ compared with fructose, suggesting an additional layer of carbon‐ or energy source‐dependent regulation beyond the transcriptional regulator (Figure 3). The deletion mutant Δ*cutMSL1*, lacking the structural subunits of CODH I, showed no CO oxidation activity, suggesting that CODH II does not contribute to CO oxidation (Figure 5B). However, methylene blue may not serve as a suitable artificial electron acceptor for CODH II, as previously described for the CODH of *Bradyrhizobium japonicum* USDA 110, which grows on CO and has only Form II CODH (Lorite et al. 2000; King and Weber 2007). Thus, the function of *H. pseudoflava* CODH II remains unclear. It is generally assumed that Form II of CODHs may exhibit a higher affinity for CO, or that CO may not represent its primary physiological substrate (King 2003; King and Weber 2007). In contrast to the CODH I cluster, we could not identify a CO‐sensing transcriptional regulator in the CODH II cluster, although the abundance of proteins in this cluster increased moderately in the presence of CO. However, this increase was several‐fold lower than that of proteins belonging to CODH I (Figure 3).

The optional heterotrophic, mixotrophic or chemolithoautotrophic lifestyle of carboxydotrophic *Knallgas* bacteria demands a highly flexible and complex energy metabolism. Figure 6 illustrates a schematic model of the chemolithoautotrophic metabolism in *H. pseudoflava* . Electrons derived from H_2_ via MBH or from CO via CODH enter the quinone pool but cannot directly reduce NAD(P)^+^ (Meyer and Schlegel 1979). It is therefore likely that in *H. pseudoflava* , NADH is generated via RET through complex I (NADH dehydrogenase), which is subsequently converted to NADPH by the transhydrogenase PntAB. Both RET and ATP synthesis rely on PMF generated by the branched respiratory chain. In *A. carboxidovorans* OM5, the CO‐sensitive branch consists of cytochromes *b*_*558*_, *c* and *a*_*1*_ (Cypionka and Meyer 1983a). Experiments with fructose have shown that *C. necator* H16 favours the energy‐efficient pathway via the cytochrome *bc*_*1*_ complex, which transfers two electrons from ubiquinol to a cytochrome *c* carrier, then to the low O_2_ affinity terminal cytochrome *aa*_*3*_ oxidase, translocating a total of six protons per ubiquinol (Jahn et al. 2024). A similar route may operate in *H. pseudoflava* under heterotrophic conditions. Our proteomics data show that the abundance of the *bc*_*1*_ complex (PetABC) did not change significantly across all conditions (Table S3). Putative *c*‐type cytochromes (*c2* and *c‐555*) and the *aa*_*3*_ complex (CtaCDE) were least abundant under CO (Figure 2, Table S3). This suggests that the pathway is preferred in the absence of CO, supported by the H^+^/O ratio (5–6) determined by Cypionka et al. (1984). In contrast to fructose‐grown cells (21% O_2_), CtaB and CtaG decreased under CO_2_/H_2_ and CtaG even under the tested mixotrophic condition (4% O_2_). Furthermore, the high O_2_ affinity complex *cbb*_*3*_ (CcoPQON) was more abundant under both conditions, which may reflect lower oxygen availability (Bueno et al. 2012; Siebert et al. 2020). It may also indicate changes in electron routing through terminal oxidases under H_2_ atmosphere, also supported by the increased abundance of cytochrome *bo*_*3*_ ubiquinol oxidase subunits (CyoABCD) (Figure 2). For example, mutants lacking SH in *C. necator* H16 that rely on NADH generation via RET exhibit a shift in the dominant electron‐transport branches (Kömen et al. 1992).

**FIGURE 6:**
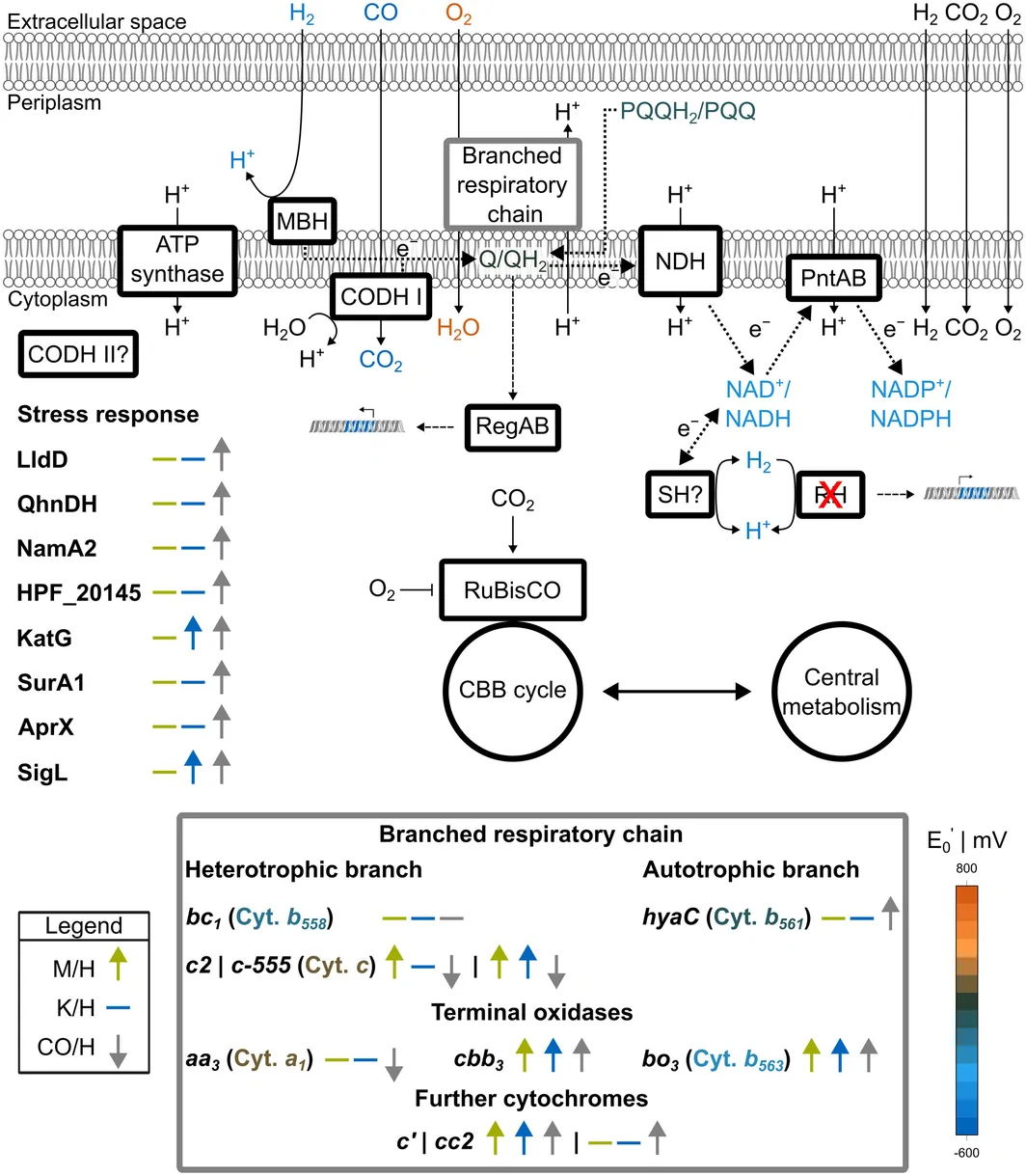
Schematic model of the chemolithoautotrophic metabolism with proposed cytochrome role assignments and putative key enzymes. Cytochrome names in parentheses refer to the branched respiratory chain model of *Afipia carboxidovorans* OM5 (Cypionka and Meyer 1983a). The figure shows the midpoint potentials (E_0_^′^) of the redox couples (Cypionka et al. 1985; Meyer et al. 1986; Akagawa et al. 2016), and protein abundance changes under mixotrophic (M, green) and chemolithoautotrophic conditions (K, blue; CO, grey) relative to heterotrophic growth (H). Higher‐abundance proteins compared to the reference condition (H) are indicated by ↑ and lower‐abundance proteins by **↓**, whereas − denotes no differential abundance. For protein complexes, the arrows represent the overall trend in subunit abundance changes. Thresholds for comparisons: Unique number of peptides ≥ 2, *p*‐value ≤ 0.05, *q*‐value ≤ 0.01, FC ≤ 0.667 and ≥ 1.5 (|log_2_FC| ≥ 0.585). Proteome samples were obtained from biological replicates (*n* = 5). *aa*_*3*_, cytochrome *aa*_*3*_ oxidase; AprX, serine protease AprX; *bc*_*1*_, *bc*_*1*_ cytochrome reductase; *bo*_*3*_, cytochrome *bo*_*3*_ ubiquinol oxidase; *c'*, cytochrome *c'*; *c2*, cytochrome *c2*; *c‐555*, cytochrome *c‐555* precursor; CBB, Calvin‐Benson‐Bassham cycle; *cbb*_*3*_, cytochrome *cbb*_*3*_ oxidase; *cc2*, cytochrome *c4* precursor; CODH, CO dehydrogenase; Cyt., cytochrome; HPF_20145, heme peroxidase; *hyaC*, putative [NiFe]‐hydrogenase 1 *b*‐type cytochrome subunit; KatG, catalase‐peroxidase; LldD, l‐lactate dehydrogenase [cytochrome]; MBH, membrane‐bound hydrogenase; NamA2, NADPH dehydrogenase; NDH, NADH‐quinone oxidoreductase; PntAB, membrane‐bound transhydrogenase; PQQ/PQQH_2_, oxidized/reduced form of pyrroloquinoline quinone; Q/QH_2_, oxidized/reduced form of ubiquinone; QhnDH, quinohemoprotein amine dehydrogenase; RegAB, two‐component regulatory system; RH, regulatory hydrogenase system; RuBisCO, ribulose 1,5‐bisphosphate carboxylase‐oxygenase; SH, soluble hydrogenase; SigL, ECF RNA polymerase sigma factor SigL; SurA1, chaperone SurA precursor. Additional details on protein annotation and proteomics data are provided in Table S3.

Under CO conditions, *A. carboxidovorans* OM5 channels electrons through the less energy‐conserving, CO‐insensitive respiratory chain branch composed of *b*_*561*_ and *b*_*563*_ (cytochrome *o*) (Cypionka and Meyer 1983a; Cypionka et al. 1984). In *H. pseudoflava* , a *b*‐type cytochrome, annotated as *hyaC* (HPF_02100), may correspond to the proposed *b*_*561*_ cytochrome and was most abundant under CO (Table S3). Notably, two *c*‐type cytochromes (*c'*, *cc2*) were significantly more abundant under CO, although their physiological function is uncharacterized (Table S3). Our CO experiments also revealed a particularly high abundance of the *bo*_*3*_ complex (Figure 2). In vitro, the *E. coli* K‐12 cytochrome *bo*_*3*_ oxidase is less sensitive to CO inhibition than *bd*‐type oxidases (Forte et al. 2019). However, in vivo data did not support this observation, identifying the cytochrome *bd*‐I ubiquinol oxidase as the primary contributor to CO tolerance (Nastasi et al. 2023, 2024). In *M. smegmatis* mc^2^155, the *bd*‐type oxidase was also characterized as CO‐resistant (Bayly et al. 2021), and in *C. necator* H16, a mutation upstream of the cytochrome *bd* oxidase operon upregulated the transcription, resulting in increased CO tolerance (Wickham‐Smith et al. 2023). No putative *bd*‐I sequence was found in the genome of *H. pseudoflava* . Thus, the *bo*_*3*_ complex may serve as the CO‐insensitive terminal oxidase of the autotrophic branch. Alternatively, the *cbb*_*3*_ complex proteins increased under CO (4% O_2_) relative to fructose (21% O_2_), though less than under CO_2_/H_2_ conditions (Figure 2). The high O_2_ affinity described for the CO‐insensitive oxidase in *A. carboxidovorans* OM5 (Cypionka et al. 1985) would be consistent with a potential role of the *cbb*_*3*_ complex as the terminal oxidase of the autotrophic branch.

Although CO oxidation yields more energy than H_2_ oxidation (ΔG°′ = −257 vs. −237 kJ mol^−1^ (Kohtz et al. 2026)), *H. pseudoflava* grew more slowly under comparable high CO concentrations (0.03 vs. 0.14 h^−1^). During growth on syngas, it exhibited fivefold higher biomass‐specific CO than H_2_ uptake rates (Grenz et al. 2019), indicating not only potential MBH inhibition (Lamle et al. 2005; Kohtz et al. 2026) but also continuous CO oxidation. Accordingly, growth on syngas was approximately twofold slower than under CO‐deficient chemolithoautotrophic conditions, with reported growth rates of 0.07 h^−1^ (20% CO; Miserez et al. 2026) and 0.06 h^−1^ (40% CO; Grenz et al. 2019). This suggests that although *H. pseudoflava* tolerates CO concentrations of up to 90% in the gas phase (Cypionka and Meyer 1982), the presence of CO imposes a metabolic burden, in addition to the less efficient autotrophic branch of the respiratory chain and the differences in PMF generation resulting from cytoplasmic CO oxidation compared to periplasmic H_2_ oxidation. Under chemolithoautotrophic conditions with unphysiologically high CO concentrations, CO oxidation may lead to a highly reduced quinone pool and, consequently, a disturbed redox balance. Furthermore, as the CO_2_ concentration during CO cultivation was unknown, the resulting CO_2_/O_2_ ratio may have influenced RuBisCO, including its oxygenase activity and the associated production and recycling of phosphoglycolate (Claassens et al. 2020). This may have affected Calvin cycle flux and the cellular NADH balance. Our proteomics data showed increased abundance of several dehydrogenases (log_2_FC ≥ 1.67) and proteins involved in PQQ biosynthesis (log_2_FC ≥ 1.13), which may support redox homeostasis (Table S3). Besides being a cofactor for dehydrogenases (Salisbury et al. 1979), PQQ also functions as an antioxidant (Misra et al. 2004). Consistent with a perturbed redox state, protein levels of oxidative stress‐associated enzymes such as the peroxidase KatG (Panek and O'Brian 2004) (log_2_FC = 2.19), the NADPH dehydrogenase NamA2 (Fitzpatrick et al. 2003) (log_2_FC = 1.12), and a putative heme peroxidase (log_2_FC = 4.52) increased. In addition, envelope‐associated signalling proteins (Helmann 2016; Jarick et al. 2018) and components of envelope proteostasis, including stress‐associated periplasmic proteases (De Geyter et al. 2016; Kim et al. 2021) were altered (Table S3). Collectively, these findings underline the physiological burden imposed by CO.

## 5 Conclusion

Acetogenic bacteria used for syngas fermentation rely on the highly efficient Wood–Ljungdahl pathway for carbon fixation, but anaerobic energy conservation remains thermodynamically constrained. In contrast, the aerobic oxidation of CO provides a greater free‐energy yield, theoretically enabling higher ATP generation. This advantage positions aerobic syngas fermentation with carboxydotrophic *Knallgas* bacteria as a complementary platform for the production of more complex chemicals. *Hydrogenophaga pseudoflava* DSM 1084 is a promising candidate for this application as it tolerates CO concentrations up to 90% and shows relatively fast growth under chemolithoautotrophic conditions with CO, CO_2_/H_2_, or their mixtures. However, the physiology of carboxydotrophic *Knallgas* bacteria—particularly their chemolithoautotrophic metabolism—remains poorly understood. Our findings advance the current understanding of the representative *H. pseudoflava* and lay the groundwork for future metabolic engineering efforts aimed at establishing aerobic syngas fermentation as a platform for the sustainable production of value‐added chemicals.

## Author Contributions

**Simon Grieshaber:** conceptualization, investigation, writing – original draft, writing – review and editing, methodology, visualization, data curation. **Antonia Ebert:** investigation, writing – review and editing, visualization, methodology, data curation. **Pauline Kehr:** investigation, writing – review and editing. **Dominik L. Siebert:** investigation, writing – review and editing. **Ammar Al‐Shameri:** writing – review and editing. **Volker Sieber:** writing – review and editing. **Birgitta E. Ebert:** writing – review and editing. **Esteban Marcellin:** writing – review and editing. **Bastian Blombach:** conceptualization, funding acquisition, writing – original draft, writing – review and editing, project administration, supervision.

## Funding

This work was supported by Bundesministerium für Bildung und Forschung (03SF0739A), Deutscher Akademischer Austauschdienst (57655196), Deutsche Forschungsgemeinschaft (BL 1408/3‐1) and Australian Research Council (IH220100012 and CE200100029).

## Conflicts of Interest

The authors declare no conflicts of interest.

## Supporting information


**Figure S1:** Growth of *Hydrogenophaga pseudoflava* WT and mutant strains under various conditions in 50 mL CO‐Ox minimal medium. (A) WT on 1% [w v^−1^] glucose, inoculated from a 1% [w v^−1^] sucrose pre‐culture. (B) Chemolithoautotrophic growth of WT (purple, circle), Δ*hyd* (blue triangle) and Δ*cbbLS* (grey, inverted triangle) under a gas atmosphere containing 10% CO_2_, 86% H_2_ and 4% O_2_. Chemolithoautotrophic growth of (C) WT (purple, circle), Δ*codh1* (blue, triangle), Δ*codh2* (grey, inverted triangle) and Δ*cbbLS* (green square) and (D) WT (purple, circle), and Δ*hoxFUYH* (blue, triangle) under a gas atmosphere containing 80% CO, 20% synthetic air. Open symbols denote time points for a complete headspace exchange. Data represents the mean, and error bars indicate the standard deviation of biological replicates (*n* ≥ 3).
**Figure S2:** Principal component analysis showing the clustering of biological replicates (*n* = 5) under the same condition and highlighting condition‐dependent differences in the global proteomic profile. Growth of *Hydrogenophaga pseudoflava* WT in 50 mL CO‐Ox minimal medium under the following conditions: H: 1% [w v^−1^] fructose (purple, circle); M: 1% [w v^−1^] fructose with a gas mixture of 10% CO_2_, 86% H_2_ and 4% O_2_ (green, triangle); K: only the CO_2_/H_2_/O_2_ gas mixture (blue, inverted triangle); CO: gas mixture of 80% CO and 20% synthetic air (grey, square).
**Figure S3:** Volcano‐plots—(A) M/H, (B) K/H, (C) CO/H—of the 3640 quantified proteins (unique number of peptides ≥ 2) in *Hydrogenophaga pseudoflava* WT, comparing the cultivation conditions M (1% [w v^−1^] fructose and a gas atmosphere of 10% CO_2_, 86% H_2_ and 4% O_2_), K (only the CO_2_/H_2_/O_2_ gas mixture) and CO (80% CO and 20% synthetic air) with H (1% [w v^−1^] fructose). Thresholds for comparisons: *p*‐value ≤ 0.05, fold change (FC) ≤ 0.667 and ≥ 1.5 (|log_2_FC| ≥ 0.585). Proteome samples were obtained from biological replicates (*n* = 5).
**Figure S4:** Investigation of the soluble hydrogenase of *Hydrogenophaga pseudoflava*. (A) Hydrogenase activity in cell‐free extract was monitored via NAD^+^ reduction at 365 nm. Absorption curves with (blue) and without (grey) H_2_ supply. *H. pseudolfava* (pOCEx1‐*Strep*II‐*hoxFUYHW*) was cultivated in 200 mL CO‐Ox minimal medium with 1% [w v^−1^] fructose. (B) Chemolithoautotrophic growth of *H. pseudoflava* Δ*hoxKG* (pOCEx1‐*Strep*II‐*hoxFUYHW*) in 50 mL CO‐Ox minimal medium cultivated under a gas atmosphere containing 10% CO_2_, 86% H_2_ and 4% O_2_. Open symbols denote time points for a complete headspace exchange. Data represents the mean, and error bars indicate the standard deviation of biological replicates (*n* = 3).
**Figure S5:** Growth of *Hydrogenophaga pseudoflava* Δ*pntAB* in comparison to the WT in 50 mL CO‐Ox minimal medium. (A) Heterotrophic growth (1% [w v^−1^] fructose) and (B) chemolithoautotrophic growth (80% CO and 20% synthetic air) of the WT (blue, square) and Δ*pntAB* (grey, triangle). (C) Chemolithoautotrophic growth (10% CO_2_, 86% H_2_, 4% O_2_) of WT (dark blue, square) and Δ*pntAB* (dark grey, triangle) in standard minimal medium and WT red (light blue, diamond) and Δ*pntAB* red (purple, circle) with reduced supplements (10% of their respective standard concentrations). (D) Chemolithoautotrophic growth (10% CO_2_, 86% H_2_, 4% O_2_) of the Δ*pntAB* mutant under individual supplement reductions: All supplements (purple, circle); MgSO_4_ (dark grey, triangle); CaCl_2_ (light grey, inverted triangle); ammonium iron(III) citrate (light blue, square); SL‐6 (dark blue, diamond). Open symbols denote time points for a complete headspace exchange. Data represents the mean, and error bars indicate the standard deviation of biological replicates (*n* ≥ 3).
**Table S1:** Strains and plasmids used in this study.
**Table S2:** Oligonucleotides used in this study.
**Table S3:** Protein abundance changes under various conditions.

**File S1:** Candidates identified in the proteomics study.

## Data Availability

The mass spectrometry proteomic data have been deposited to the ProteomeXchange Consortium via the PRIDE (Perez‐Riverol et al. 2025) partner repository, with the dataset identifier PXD068401.
