# Supplementary material for: Unravelling the Chemolithoautotrophic Metabolism of the Carboxydotrophic Knallgas Bacterium Hydrogenophaga pseudoflava
Source: Microb Biotechnol. 2026 Nov 1;19(11):e70452. doi: 10.1111/1751-7915.70452 (PMC13725076; doi:10.1111/1751-7915.70452)
Supplement: Supplementary file 1 [file MBT2-19-e70452-s002.zip › 1_FigureS1.pdf]

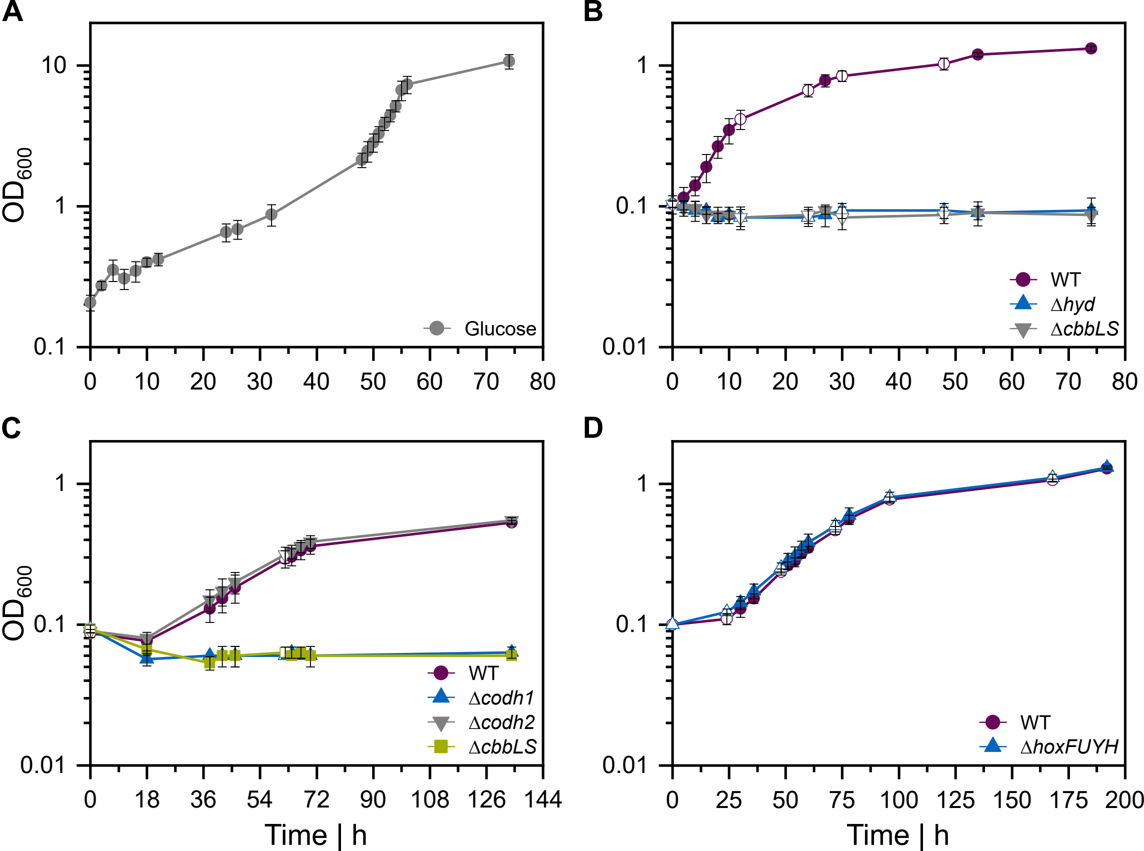

A
B
C
D
OD$_{600}$
Time | h
Glucose
WT
Δhyd
ΔcbbLS
WT
Δcodh1
Δcodh2
ΔcbbLS
WT
ΔhoxFUYH
