# Supplementary material for: Unravelling the Chemolithoautotrophic Metabolism of the Carboxydotrophic Knallgas Bacterium Hydrogenophaga pseudoflava
Source: Microb Biotechnol. 2026 Nov 1;19(11):e70452. doi: 10.1111/1751-7915.70452 (PMC13725076; doi:10.1111/1751-7915.70452)
Supplement: Supplementary file 1 [file MBT2-19-e70452-s002.zip › 1_FigureS2.pdf]

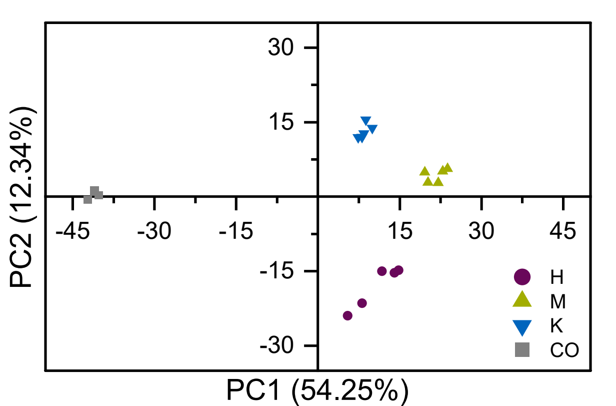

30
15
-15
-30
-45
-30
-15
15
30
45
PC2 (12.34%)
PC1 (54.25%)
H
M
K
CO
