# Supplementary material for: Unravelling the Chemolithoautotrophic Metabolism of the Carboxydotrophic Knallgas Bacterium Hydrogenophaga pseudoflava
Source: Microb Biotechnol. 2026 Nov 1;19(11):e70452. doi: 10.1111/1751-7915.70452 (PMC13725076; doi:10.1111/1751-7915.70452)
Supplement: Supplementary file 1 [file MBT2-19-e70452-s002.zip › 1_FigureS3.pdf]

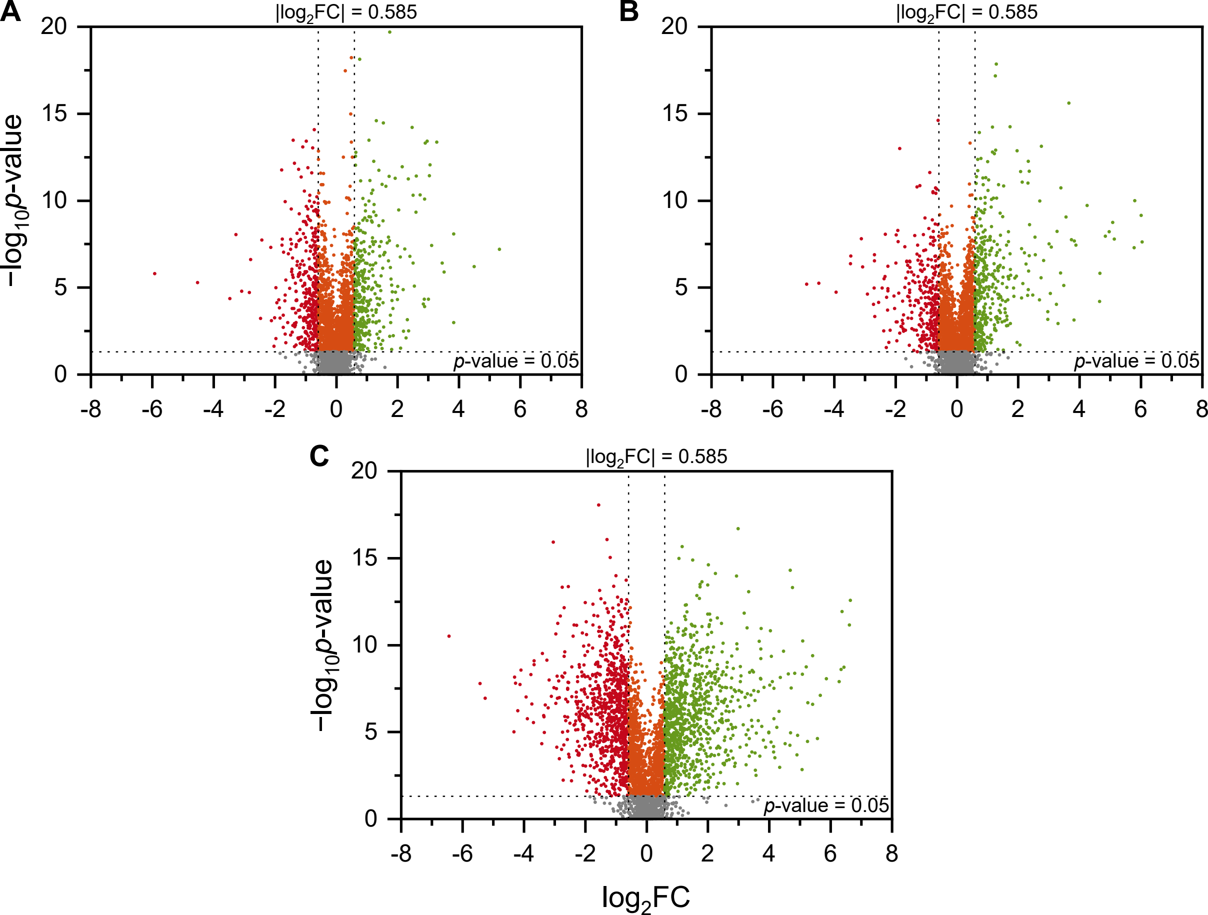
A
B
C
|log2FC| = 0.585
p-value = 0.05
-log10p-value
log2FC
20
15
10
5
0
-8
-6
-4
-2
0
2
4
6
8
