# Supplementary material for: Unravelling the Chemolithoautotrophic Metabolism of the Carboxydotrophic Knallgas Bacterium Hydrogenophaga pseudoflava
Source: Microb Biotechnol. 2026 Nov 1;19(11):e70452. doi: 10.1111/1751-7915.70452 (PMC13725076; doi:10.1111/1751-7915.70452)
Supplement: Supplementary file 1 [file MBT2-19-e70452-s002.zip › 1_FigureS4.pdf]

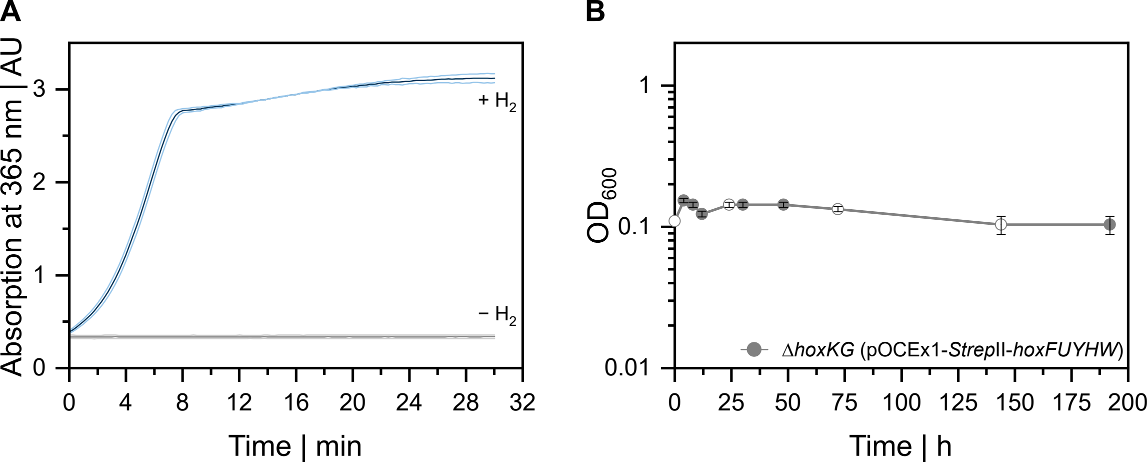

A
Absorption at 365 nm | AU
0
1
2
3
+ H2
− H2
0
4
8
12
16
20
24
28
32
Time | min
B
OD600
0.01
0.1
1
ΔhoxKG (pOCEx1-StrepII-hoxFUYHW)
0
25
50
75
100
125
150
175
200
Time | h
