# Supplementary material for: Unravelling the Chemolithoautotrophic Metabolism of the Carboxydotrophic Knallgas Bacterium Hydrogenophaga pseudoflava
Source: Microb Biotechnol. 2026 Nov 1;19(11):e70452. doi: 10.1111/1751-7915.70452 (PMC13725076; doi:10.1111/1751-7915.70452)
Supplement: Supplementary file 1 [file MBT2-19-e70452-s002.zip › 1_FigureS5.pdf]

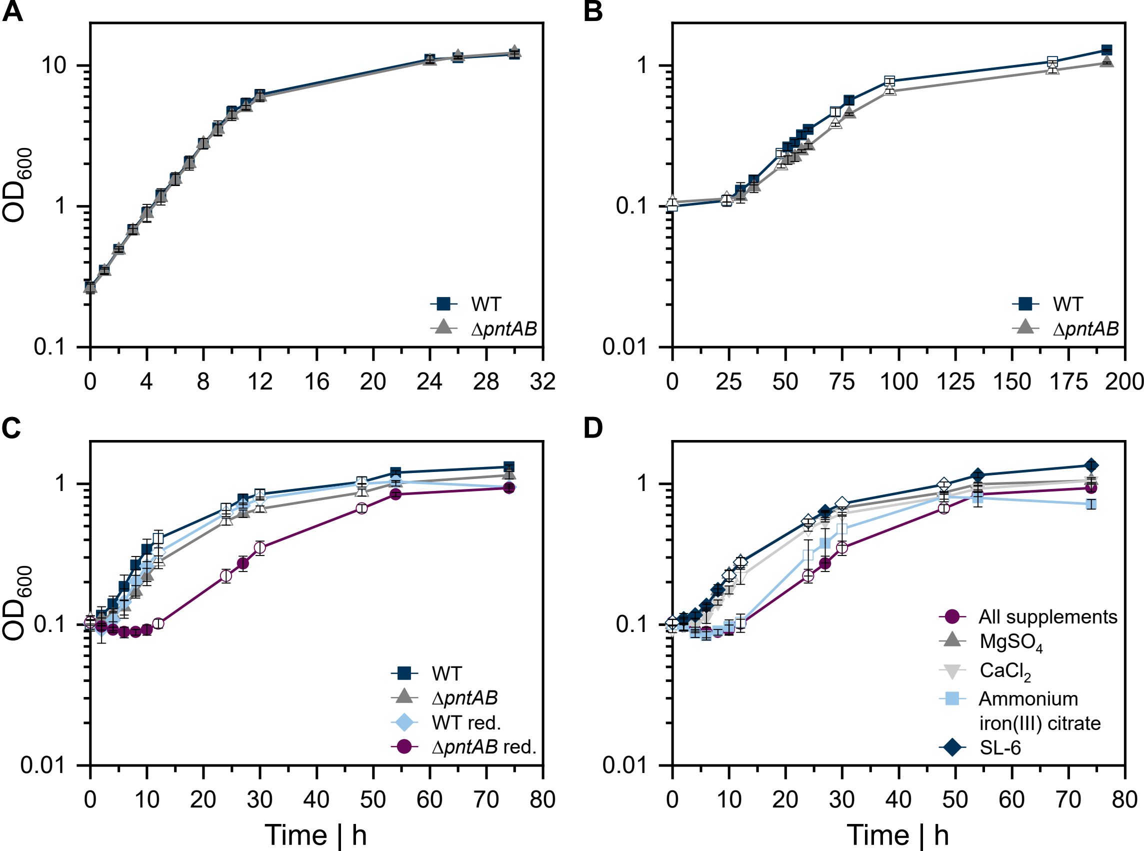
A
B
C
D
OD600
Time | h
WT
ΔpntAB
WT red.
ΔpntAB red.
All supplements
MgSO4
CaCl2
Ammonium
iron(III) citrate
SL-6
0.01
0.1
1
10
0
4
8
12
16
20
24
28
32
25
50
75
100
125
150
175
200
10
20
30
40
50
60
70
80
