# Supplementary material for: Unravelling the Chemolithoautotrophic Metabolism of the Carboxydotrophic Knallgas Bacterium Hydrogenophaga pseudoflava
Source: Microb Biotechnol. 2026 Nov 1;19(11):e70452. doi: 10.1111/1751-7915.70452 (PMC13725076; doi:10.1111/1751-7915.70452)
Supplement: Supplementary file 1 [file MBT2-19-e70452-s002.zip › Grieshaber et al. 2026_Supplements.docx]

**Supplemental information**

**Unravelling the Chemolithoautotrophic Metabolism of the Carboxydotrophic *Knallgas* Bacterium *Hydrogenophaga pseudoflava***

**Simon Grieshaber^1^, Antonia Ebert^2,3,4^, Pauline Kehr^1^, Dominik L. Siebert^5^, Ammar Al-Shameri^5^, Volker Sieber^5,6,7^, Birgitta E. Ebert^2^, Esteban Marcellin^2^, Bastian Blombach^1,7,*^**

^1^Microbial Biotechnology, Campus Straubing for Biotechnology and Sustainability, Technical University of Munich, Straubing, Germany.

^2^Australian Institute for Bioengineering and Nanotechnology, The University of Queensland, Brisbane, Queensland, Australia.

^3^ARC Centre of Excellence in Synthetic Biology, The University of Queensland, Brisbane, Queensland, Australia

^4^Advanced Engineering Biology Future Science Platform, CSIRO, Brisbane, Queensland, Australia

^5^Chair of Chemistry of Biogenic Resources, Campus Straubing for Biotechnology and Sustainability, Technical University of Munich, Straubing, Germany.

^6^Catalytic Research Center, Technical University of Munich, Garching, Germany.

^7^SynBiofoundry@TUM, Technical University of Munich, Straubing, Germany.

*Corresponding author: Bastian Blombach (bastian.blombach@tum.de), Microbial Biotechnology, Campus Straubing for Biotechnology and Sustainability, Technical University of Munich, Uferstraße 53, 94315 Straubing, Germany; https://orcid.org/0000-0002-2996-2049

Keywords:

aerobic gas fermentation | branched respiratory chain | carbon dioxide | carbon monoxide | chemolithoautotrophic metabolism | CO dehydrogenase | hydrogenase | *Hydrogenophaga pseudoflava* | proteomics | transhydrogenase

Figure S1: Growth of *Hydrogenophaga pseudoflava* WT and mutant strains under various conditions in 50 mL CO-Ox minimal medium. (A) WT on 1% [w v^−1^] glucose, inoculated from a 1% [w v^−1^] sucrose pre-culture. (B) Chemolithoautotrophic growth of WT (purple, circle), Δ*hyd* (blue triangle), and Δ*cbbLS* (grey, inverted triangle) under a gas atmosphere containing 10% CO_2_, 86% H_2_, and 4% O_2_. Chemolithoautotrophic growth of (C) WT (purple, circle), Δ*codh1* (blue, triangle), Δ*codh2* (grey, inverted triangle), and Δ*cbbLS* (green square), and (D) WT (purple, circle), and Δ*hoxFUYH* (blue, triangle) under a gas atmosphere containing 80% CO, 20% synthetic air. Open symbols denote time points for a complete headspace exchange. Data represents the mean, and error bars indicate the standard deviation of biological replicates (*n* ≥ 3).

Figure S2: Principal component analysis showing the clustering of biological replicates (*n* = 5) under the same condition and highlighting condition-dependent differences in the global proteomic profile. Growth of *Hydrogenophaga pseudoflava* WT in 50 mL CO-Ox minimal medium under the following conditions: H: 1% [w v^−1^] fructose (purple, circle); M: 1% [w v^−1^] fructose with a gas mixture of 10% CO_2_, 86% H_2_, and 4% O_2_ (green, triangle); K: only the CO_2_/H_2_/O_2_ gas mixture (blue, inverted triangle); CO: gas mixture of 80% CO and 20% synthetic air (grey, square).

Figure S3: Volcano-plots–(A) M/H, (B) K/H, (C) CO/H–of the 3640 quantified proteins (unique number of peptides ≥ 2) in *Hydrogenophaga pseudoflava* WT, comparing the cultivation conditions M (1% [w v^−1^] fructose and a gas atmosphere of 10% CO_2_, 86% H_2_, and 4% O_2_), K (only the CO_2_/H_2_/O_2_ gas mixture), and CO (80% CO and 20% synthetic air) with H (1% [w v^−1^] fructose). Thresholds for comparisons: p-value ≤ 0.05, fold change (FC) ≤ 0.667 and ≥ 1.5 (|log_2_FC| ≥ 0.585). Proteome samples were obtained from biological replicates (*n* = 5).

Figure S4: Investigation of the soluble hydrogenase of *Hydrogenophaga pseudoflva.* (A) Hydrogenase activity in cell-free extract was monitored via NAD^+^ reduction at 365 nm. Absorption curves with (blue) and without (grey) H_2_ supply. *H. pseudolfava* (pOCEx1-*Strep*II-*hoxFUYHW*) was cultivated in 200 mL CO-Ox minimal medium with 1% [w v^−1^] fructose. (B) Chemolithoautotrophic growth of *H. pseudoflava* Δ*hoxKG* (pOCEx1-*Strep*II-*hoxFUYHW*) in 50 mL CO-Ox minimal medium cultivated under a gas atmosphere containing 10% CO_2_, 86% H_2_, and 4% O_2_. Open symbols denote time points for a complete headspace exchange. Data represents the mean, and error bars indicate the standard deviation of biological replicates (*n* = 3).

Figure S5: Growth of *Hydrogenophaga pseudoflava* Δ*pntAB* in comparison to the WT in 50 mL CO-Ox minimal medium. (A) Heterotrophic growth (1% [w v^−1^] fructose) and (B) chemolithoautotrophic growth (80% CO and 20% synthetic air) of the WT (blue, square) and Δ*pntAB* (grey, triangle). (C) Chemolithoautotrophic growth (10% CO_2_, 86% H_2_, 4% O_2_) of WT (dark blue, square) and Δ*pntAB* (dark grey, triangle) in standard minimal medium, and WT red. (light blue, diamond) and Δ*pntAB* red. (purple, circle) with reduced supplements (10% of their respective standard concentrations). (D) Chemolithoautotrophic growth (10% CO_2_, 86% H_2_, 4% O_2_) of the Δ*pntAB* mutant under individual supplement reductions: All supplements (purple, circle); MgSO_4_ (dark grey, triangle); CaCl_2_ (light grey, inverted triangle); ammonium iron(III) citrate (light blue, square); SL-6 (dark blue, diamond). Open symbols denote time points for a complete headspace exchange. Data represents the mean, and error bars indicate the standard deviation of biological replicates (*n* ≥ 3).

**Table S1:** Strains and plasmids used in this study.

| No. | Strain or plasmid | Relevant characteristics | Reference |
| --- | --- | --- | --- |
| **Strains** | | | |
| S1 | *Escherichia coli* S17-1 | t*hi* *pro hsdR^−^* *hsdM^+^* *recA* RP4-2-Tc::Mu-Km::Tn7 | Simon *et al.* (1983) |
| S2 | *Hydrogenophaga pseudoflava* DSM 1084 | Wild type | Zavarzin and Nozhevnikova (1977) |
| S3 | *H. pseudoflava* Δ*cutMSL1* | *H. pseudoflava* DSM 1084 derivative with deletion of the genes *cutM* (HPF_04405), *cutS* (HPF_04400), and *cutL1* (HPF_04395), encoding the structural subunits of the CO dehydrogenase 1 | This study |
| S4 | *H. pseudoflava* Δ*coxSL2M* | *H. pseudoflava* DSM 1084 derivative with deletion of the genes *coxS* (HPF_17415), *cutL2* (HPF_17420), and *coxM* (HPF_17425), encoding the structural subunits of the CO dehydrogenase 2 | This study |
| S5 | *H. pseudoflava* Δ*codh1* | *H. pseudoflava* DSM 1084 derivative with deletion of the CO dehydrogenase cluster 1 encoding genes (HPF_04410–HPF_04350) | This study |
| S6 | *H. pseudoflava* Δ*codh2* | *H. pseudoflava* DSM 1084 derivative with deletion of the CO dehydrogenase cluster 2 encoding genes (HPF_17415–HPF_17445) | This study |
| S7 | *H. pseudoflava* Δ*hoxFUYH* | *H. pseudoflava* DSM 1084 derivative with deletion of the genes *hoxF* (HPF_01495), *hoxU* (HPF_01500), *hoxY* (HPF_01505), and *hoxH* (HPF_01510), encoding the structural subunits of the soluble hydrogenase | This study |
| S8 | *H. pseudoflava* Δ*hoxKG* | *H. pseudoflava* DSM 1084 derivative with deletion of the genes *hoxK* (HPF_01390), and *hoxG* (HPF_01395), encoding the structural subunits of the membrane-bound hydrogenase | This study |
| S9 | *H. pseudoflava* Δ*hoxBCJ12* | *H. pseudoflava* DSM 1084 derivative with deletion of the genes *hoxB* (HPF_01520), *hoxC* (HPF_01525), *hoxJ1* (HPF_01530), and *hoxJ2* (HPF_01535), encoding the structural subunits of the regulatory hydrogenase and histidine protein kinase HoxJ | This study |
| S10 | *H. pseudoflava* Δ*hoxA* | *H. pseudoflava* DSM 1084 derivative with deletion of the gene *hoxA* (HPF_01490), encoding the hydrogenase transcriptional regulatory protein | This study |
| S11 | *H. pseudoflava* Δ*hyd* | *H. pseudoflava* DSM 1084 derivative with deletion of the hydrogenase cluster encoding genes (HPF_01390–HPF_01535) | This study |
| S12 | *H. pseudoflava* Δ*pntAB* | *H. pseudoflava* DSM 1084 derivative with deletion of the genes *pntA* (HPF_21995), and *pntB* (HPF_21990), encoding the structural subunits of the transhydrogenase | This study |
| S13 | *H. pseudoflava* Δ*cbbLS* | *H. pseudoflava* DSM 1084 derivative with deletion of the genes *cbbL* (HPF_05955), and *cbbS* (HPF_05960), encoding the structural subunits of the RuBisCO | This study |

**Table S1:** Strains and plasmids used in this study, continued.

| S14 | *H. pseudoflava* (pOCEx1-*Strep*II-*hoxFUYHW*) | *H. pseudoflava* DSM 1084 derivative carrying the plasmid pOCEx1-*Strep*II-*hoxFUYHW* | Siebert *et al.* (2026) |
| --- | --- | --- | --- |
| S15 | *H. pseudoflava* Δ*hoxKG* (pOCEx1-*Strep*II-*hoxFUYHW*) | *H. pseudoflava* DSM 1084 derivative with deletion of the genes *hoxK* (HPF_01390), and *hoxG* (HPF_01395), encoding the structural subunits of the membrane-bound hydrogenase, carrying the plasmid pOCEx1-*Strep*II-*hoxFUYHW* | This study |
| **Plasmids** | | | |
| P1 | pK19*mobsacB* | Km^R^, *sacB,* *lacZα,* oriV_pMB1_, oriT_RP4_, | Schäfer *et al.* (1994) |
| P2 | pK19*mobsacB-*Δ*cutMSL1* | pK19*mobsacB* derivative for the deletion of the genes *cutM*, *cutS*, and *cutL1* | This study |
| P3 | pK19*mobsacB-*Δ*coxSL2M* | pK19*mobsacB* derivative for the deletion of the genes *coxS*, *cutL2*, and *coxM* | This study |
| P4 | pK19*mobsacB-*Δ*codh1* | pK19*mobsacB* derivative for the deletion of the CO dehydrogenase cluster 1 | This study |
| P5 | pK19*mobsacB-*Δ*codh2* | pK19*mobsacB* derivative for the deletion of the CO dehydrogenase cluster 2 | This study |
| P6 | pK19*mobsacB-*Δ*hoxFUYH* | pK19*mobsacB* derivative for the deletion of the genes *hoxF*, *hoxU*, *hoxY*, and *hoxH* | This study |
| P7 | pK19*mobsacB-*Δ*hoxKG* | pK19*mobsacB* derivative for the deletion of the genes *hoxK* and *hoxG* | This study |
| P8 | pK19*mobsacB-*Δ*hoxBCJ12* | pK19*mobsacB* derivative for the deletion of the genes *hoxB*, *hoxC*, *hoxJ1*, and *hoxJ2* | This study |
| P9 | pK19*mobsacB-*Δ*hoxA* | pK19*mobsacB* derivative for the deletion of the gene *hoxA* | This study |
| P10 | pK19*mobsacB-*Δ*hyd* | pK19*mobsacB* derivative for the deletion of the hydrogenase cluster | This study |
| P11 | pK19*mobsacB-*Δ*pntAB* | pK19*mobsacB* derivative for the deletion of the genes *pntA* and *pntB* | This study |
| P12 | pK19*mobsacB-*Δ*cbbLS* | pK19*mobsacB* derivative for the deletion of the genes *cbbL* and *cbbS* | This study |
| P13 | pOCEx1 | Km^R^, *lacZα*, *lacI^q^*, P*_tac_*, oriV_pBBR1_, oriT_RK2_ | Grenz *et al.* (2019) |
| P14 | pOCEx1-*Strep*II-*hoxFUYHW* | pOCEx1 derivative for the overexpression of the soluble hydrogenase. Artificial ribosomal binding site with a spacer sequence (Siebert *et al.* 2020) is followed by the start codon ATG and a *Strep*-tag II (Schmidt and Skerra 2007) linked to hoxF_ΔATG_. Plasmid includes genes *hoxF*, *hoxU*, *hoxY*, and *hoxH* encoding the structural subunits and the endopeptidase HoxW (HPF_01515) | Siebert *et al.* (2026) |

**Table S2:** Oligonucleotides used in this study.

| No. | Primer | Sequence (5’ → 3’) | Purpose |
| --- | --- | --- | --- |
| 1 | pK19_lacI_fw | TCAGTGAGCGAGGAAGCG | To verify pK19*mobsacB* derivates via PCR/sequencing  Werner *et al.* (2023) |
| 2 | pK19_lacZ_rv | TTAGCAGCCCTTGCGCC | To verify pK19*mobsacB* derivates via PCR/sequencing  Werner *et al.* (2023) |
| 3 | lacZ_fw | ATGACCATGATTACGCCAAGCG | To verify pOCEx1 derivatives via PCR/sequencing  Siebert *et al.* (2020) |
| 4 | lacZ_rv | TTACAATTTCCATTCGCCATTCAGG | To verify pOCEx1 derivatives via PCR/sequencing  Siebert *et al.* (2020) |
| 5 | rpsM_fw | AACGCCAGACTTAAGGAATAAGTCG | To verify *E. coli* contaminations |
| 6 | rpsM_rv | CGAGCCGAAGCATAAACAGC | To verify *E. coli* contaminations |
| 7 | hin2_fw | GCATCTGTTCGAGGATCATGCTTC | To verify *H. pseudoflava* strains |
| 8 | hin2_rv | GATCAGCGTGGTTCGCTTG | To verify *H. pseudoflava* strains |
| 9 | cutMSL1_dw_fw | AGAAGACAGGAGACAACACG AGGCAAGACGATCATGGAAGTCA | To construct pK19*mobsacB_* Δ*cutMSL1* and verify via sequencing |
| 10 | cutMSL1_dw_rv | **TGAATTCGAGCTCGGTACCC** GCAGGAACTCGCGGCTG | To construct pK19*mobsacB_* Δ*cutMSL1* and verify via sequencing |
| 11 | cutMSL1_up_fw | **GTCGACTCTAGAGGATCCCC** CGTTCGGAGACAACACAGATGG | To construct pK19*mobsacB_* Δ*cutMSL1* and verify via sequencing |
| 12 | cutMSL1_up_rv | CTTCCATGATCGTCTTGCCT CGTGTTGTCTCCTGTCTTCTGG | To construct pK19*mobsacB_* Δ*cutMSL1* and verify via sequencing |
| 13 | DcutMSL1_fw | CCTGGAATCGCAGGCGC | To verify pK19*mobsacB_* Δ*cutMSL1* via sequencing and *cutMSL1* deletion by PCR |
| 14 | DcutMSL1_rv | CTGCGATCGCTTGCGAC | To verify pK19*mobsacB_* Δ*cutMSL1* via sequencing and *cutMSL1* deletion by PCR |
| 15 | codh1_dw_fw | CGCGTTCGGAGACAACACAG TGTCCGGCAACCTCTGC | To construct pK19*mobsacB_* Δ*codh1* and verify via sequencing |
| 16 | codh1_dw_rv | **TGAATTCGAGCTCGGTACCC** GAAGTCCTTGACCGATTCGCTG | To construct pK19*mobsacB*_ Δ*codh1* and verify via sequencing |
| 17 | codh1_up_fw | **GTCGACTCTAGAGGATCCCC** GCAGAGCTCGGTCTGGATCA | To construct pK19*mobsacB_* Δ*codh1* and verify via sequencing |
| 18 | codh1_up_rv | GCGGCAGAGGTTGCCGGACA CTGTGTTGTCTCCGAACGCG | To construct pK19*mobsacB*_ Δ*codh1* and verify via sequencing |

**Table S2:** Oligonucleotides used in this study, continued.

| 19 | Dcodh1_fw | AGGAACTTGTTGCCCAGGC | To verify pK19*mobsacB_* Δ*codh1* via sequencing and the *codh1* cluster deletion by PCR |
| --- | --- | --- | --- |
| 20 | Dcodh1_rv | GTCCCAGTCCACACCGAGTT | To verify pK19*mobsacB_* Δ*codh1* via sequencing and the *codh1* cluster deletion by PCR |
| 21 | coxSL2M_dw_fw | CTGGCGGTGACAGCCGGAAA CTGTGTCTCCTGATGGTTGGG | To construct pK19*mobsacB_* Δ*coxSL2M* and verify via sequencing |
| 22 | coxSL2M_up_fw | **GTCGACTCTAGAGGATCCCC** AGCACCTCGGGGTCGAC | To construct pK19*mobsacB_* Δ*coxSL2M* and verify via sequencing |
| 23 | coxSL2M_up_rv | CCAACCATCAGGAGACACAG TTTCCGGCTGTCACCGC | To construct pK19*mobsacB_* Δ*coxSL2M* and verify via sequencing |
| 24 | DcoxSL2M_fw | GAATGCTGACCTGGTACTCGC | To verify pK19*mobsacB_* Δ*coxSL2M* via sequencing and *coxSL2M* deletion by PCR |
| 25 | codh2_dw_fw | ATTTCCGAGGGCATCTTGCT CTGTGTCTCCTGATGGTTGGG | To construct pK19*mobsacB_* Δ*codh2* and verify via sequencing |
| 26 | codh2_dw_rv | **TGAATTCGAGCTCGGTACCC** GGAGGTGACGGTGCCC | To construct pK19*mobsacB_* Δ*coxSL2M* + pK19*mob-sacB_*Δ*codh2* and verify via sequencing |
| 27 | codh2_up_fw | **GTCGACTCTAGAGGATCCCC** TGCGTCACCAGGTGCTTG | To construct pK19*mobsacB_* Δ*codh2* and verify via sequencing |
| 28 | codh2_up_rv | CCAACCATCAGGAGACACAG AGCAAGATGCCCTCGGAAATC | To construct pK19*mobsacB_* Δ*codh2* and verify via sequencing |
| 29 | Dcodh2_fw | GCGGGTTGTTGCGGTC | To verify pK19*mobsacB_* Δ*codh2* via sequencing and the *codh2* cluster deletion by PCR |
| 30 | Dcodh2_rv | ATTTCGCACTGCAGGCAG | To verify pK19*mobsacB_* Δ*coxSL2M* and pK19*mob-sacB_*Δ*codh2* via sequencing and the *coxSL2M* and *codh2* cluster deletion by PCR |
| 31 | hoxFUYH_dw_fw | TGCGTCTCGCCAGTGGACGA ATGTGGGCCAGCAGTTCG | To construct pK19*mobsacB_* Δ*hoxFYUH* and verify via sequencing |
| 32 | hoxFUYH_dw_rv | **TGAATTCGAGCTCGGTACCC** CCGCGAGCCAGATGGC | To construct pK19*mobsacB_* Δ*hoxFYUH* and verify via sequencing |
| 33 | hoxFUYH_up_fw | **GTCGACTCTAGAGGATCCCC** CGTGTCGATGACCCAGCTC | To construct pK19*mobsacB_* Δ*hoxFYUH* and verify via sequencing |
| 34 | hoxFUYH_up_rv | CGCGAACTGCTGGCCCACAT TCGTCCACTGGCGAGAC | To construct pK19*mobsacB_* Δ*hoxFYUH* and verify via sequencing |

**Table S2:** Oligonucleotides used in this study, continued.

| 35 | DhoxFUYH_fw | GCCACAGGACGTTCATGC | To verify pK19*mobsacB_* Δ*hoxFUYH* via sequencing and *hoxFUYH* deletion by PCR |
| --- | --- | --- | --- |
| 36 | DhoxFUYH_rv | GAGGGCCTGTTCCGCG | To verify pK19*mobsacB_* Δ*hoxFUYH* via sequencing and *hoxFUYH* deletion by PCR |
| 37 | hoxKG_dw_fw | CGTGACCTTGGCCATCTCCT GAAACTGCGGCGCGAAATG | To construct pK19*mobsacB_* Δ*hoxKG* and verify via sequencing |
| 38 | hoxKG_dw_rv | **TGAATTCGAGCTCGGTACCC** ACCGGTATCACCAGCGACT | To construct pK19*mobsacB_* Δ*hoxKG* and verify via sequencing |
| 39 | hoxKG_up_fw | **GTCGACTCTAGAGGATCCCC** ATGGTGGTTGCCCACGATG | To construct pK19*mobsacB_* Δ*hoxKG* and verify via sequencing |
| 40 | hoxKG_up_rv | GCATTTCGCGCCGCAGTTTC AGGAGATGGCCAAGGTCAC | To construct pK19*mobsacB_* Δ*hoxKG* and verify via sequencing |
| 41 | DhoxKG_fw | AGCACCACGCTCAAAGAGATG | To verify pK19*mobsacB_* Δ*hoxKG* via sequencing and *hoxKG* deletion by PCR |
| 42 | DhoxKG_rv | GCTGCATTTCCTGTGCCAC | To verify pK19*mobsacB_* Δ*hoxKG* and pK19*mobsacB_* Δ*hyd* via sequencing and *hoxKG* and *hyd* cluster deletion by PCR |
| 43 | hoxBCJ12_dw_fw | GGCAACTCGAGCACGAACTC TGGAAGTCGGTCGTGTCG | To construct pK19*mobsacB_* Δ*hoxBCJ12* and verify via sequencing |
| 44 | hoxBCJ12_dw_rv | **TGAATTCGAGCTCGGTACCC** GTCAAGAGCTGCAATCTGATCGTC | To construct pK19*mobsacB_* Δ*hoxBCJ12* and verify via sequencing |
| 45 | hoxBCJ12_up_fw | **GTCGACTCTAGAGGATCCCC** TGGAGAACCCCACCGTGG | To construct pK19*mobsacB_* Δ*hoxBCJ12* and pK19*mobsacB_*Δ*hyd* and verify via sequencing |
| 46 | hoxBCJ12_up_rv | GCCGACACGACCGACTTCCA GAGTTCGTGCTCGAGTTGCC | To construct pK19*mobsacB_* Δ*hoxBCJ12* and verify via sequencing |
| 47 | DhoxBCJ12_fw | GAGCGAGAACAGGATCAGCG | To verify pK19*mobsacB_* Δ*hoxBCJ12* and pK19*mobsacB_* Δ*hyd* via sequencing and *hoxBCJ12* and *hyd* cluster deletion by PCR |
| 48 | DhoxBCJ12_rv | CACTGTCGGTGACGCTGC | To verify pK19*mobsacB_* Δ*hoxBCJ12* via sequencing and *hoxBCJ12* deletion by PCR |
| 49 | hoxA_dw_fw | CACCATGGCCTCGATCACGT AACACGGTGAAGTCCTCGTC | To construct pK19*mobsacB_* Δ*hoxA* and verify via sequencing |
| 50 | hoxA_dw_rv | **TGAATTCGAGCTCGGTACCC** GCCAGGGCCATTTCAAACAG | To construct pK19*mobsacB_* Δ*hoxA* and verify via sequencing |

**Table S2:** Oligonucleotides used in this study, continued.

| 51 | hoxA_up_fw | **GTCGACTCTAGAGGATCCCC** TCATGCCGTCGAACAGCTC | To construct pK19*mobsacB_* Δ*hoxA* and verify via sequencing |
| --- | --- | --- | --- |
| 52 | hoxA_up_rv | GACGAGGACTTCACCGTGTT ACGTGATCGAGGCCATGGTG | To construct pK19*mobsacB_* Δ*hoxA* and verify via sequencing |
| 53 | DhoxA_fw | CAGCGGTCGACGCAGAAG | To verify pK19*mobsacB_* Δ*hoxA* via sequencing and *hoxA* deletion by PCR |
| 54 | DhoxA_rv | TGCTCAATCCGCACTACAAGAAC | To verify pK19*mobsacB_* Δ*hoxA* via sequencing and *hoxA* deletion by PCR |
| 55 | hyd_dw_fw | GGCAACTCGAGCACGAACTC CGTGTGGTGTCTCCGTGG | To construct pK19*mobsacB_* Δ*hyd* and verify via sequencing |
| 56 | hyd_dw_rv | **TGAATTCGAGCTCGGTACCC**  ACCGGTATCACCAGCGACT | To construct pK19*mobsacB_* Δ*hyd* and verify via sequencing |
| 57 | hyd_up_rv | GCCCACGGAGACACCACACG GAGTTCGTGCTCGAGTTGCC | To construct pK19*mobsacB_* Δ*hyd* and verify via sequencing |
| 58 | pntAB_dw_fw | GACCTGCCCATGACTTCTGG GACAACAACCGCATGCTGTTC | To construct pK19*mobsacB_* Δ*pntAB* and verify via sequencing |
| 59 | pntAB_dw_rv | **TGAATTCGAGCTCGGTACCC** TTGCGCACGACGTAGTTGAC | To construct pK19*mobsacB_* Δ*pntAB* and verify via sequencing |
| 60 | pntAB_up_fw | **GTCGACTCTAGAGGATCCCC** AAATTCGGCAGGGATGCGATG | To construct pK19*mobsacB_* Δ*pntAB* and verify via sequencing |
| 61 | pntAB_up_rv | GAACAGCATGCGGTTGTTGTC CCAGAAGTCATGGGCAGGTC | To construct pK19*mobsacB_* Δ*pntAB* and verify via sequencing |
| 62 | DpntAB_fw | CATGACGTACTCGGTCGAGTG | To verify pK19*mobsacB_* Δ*pntAB* via sequencing and *pntAB* deletion by PCR |
| 63 | DpntAB_rv | GTAGAGCGGGTTGACGTTGAC | To verify pK19*mobsacB_* Δ*pntAB* via sequencing and *pntAB* deletion by PCR |
| 64 | cbbLS_dw_fw | CCGTCTTCCCGCGGTAGATG CTGCATTGCTCCTGGTTGGG | To construct pK19*mobsacB_* Δ*cbbLS* and verify via sequencing |
| 65 | cbbLS_dw_rv | **TGAATTCGAGCTCGGTACCC** GTGAACAGCCAGTCCTTGAAAG | To construct pK19*mobsacB_* Δ*cbbLS* and verify via sequencing |
| 66 | cbbLS_up_fw | **GTCGACTCTAGAGGATCCCC** ACTCGGCGTGGTCGTG | To construct pK19*mobsacB_* Δ*cbbLS* and verify via sequencing |
| 67 | cbbLS_up_rv | CCCAACCAGGAGCAATGCAG CATCTACCGCGGGAAGACG | To construct pK19*mobsacB_* Δ*cbbLS* and verify via sequencing |
| 68 | DcbbLS_fw | CACGGTGGTGTCCTGGC | To verify pK19*mobsacB_* Δ*cbbLS* via sequencing and *cbbLS* deletion by PCR |

**Table S2:** Oligonucleotides used in this study, continued.

| 69 | DcbbLS_rv | CATCGCCACGTCGATGCC | To verify pK19*mobsacB_* Δ*cbbLS* via sequencing and *cbbLS* deletion by PCR |
| --- | --- | --- | --- |
| 70 | hoxF_STIIΔATG_UYHW_dw | **TTCCTGCAGCCCGGGGGATC** GAGGAGAACGGAATG GCTAGCTGGAGCCACCC GCAGTTCGAAAAAGGCGCC ACCGCCTTTGACCAGCC | To construct pOCEx1-*Strep*II_*hoxFUYHW*  Siebert *et al.* (2026) |
| 71 | hoxF_STIIΔATG_UYHW_up | **CGGCCGCTCTAGAACTAGTG** TCATGCGGGGACCTCTTCC | To construct pOCEx1-*Strep*II_*hoxFUYHW*  Siebert *et al.* (2026) |
| 72 | cutM_rv | AAGCGCAGCTTCATCATCGG | To verify *cutMSL1* and *codh1* cluster deletion by PCR |
| 73 | coxS_fw | AGCTTGCTGAAGTCGGATGC | To verify *coxSL2M* and *codh2* cluster deletion by PCR |
| 74 | hoxH_rv | TCAAGCCCTGGACCTACATGAAG | To verify *hoxFUYH* deletion by PCR |
| 75 | hoxK_fw | ATCAGGGTGTCGTCGTAGTCC | To verify *hoxKG* and *hyd* cluster deletion by PCR |
| 76 | hoxC_fw | CGAGCAGACGGGAACTCATTTG | To verify *hoxBCJ12* deletion by PCR |
| 77 | hoxA_fw | CGATGTCTCGCCGATTTCGTC | To verify *hoxA* deletion by PCR |
| 78 | pntB_rv | GGTCGTTGCTGAGCATGAAG | To verify *pntAB* deletion by PCR |
| 79 | cbbL_fw | TCTCGTCGTCCTTGGTGAAGTC | To verify *cbbLS* deletion by PCR |

*Bold sequence: overlapping plasmid sequence for Gibson assembly.
*Underlined sequence: overlapping the other flanking region of the gene for Gibson assembly.
*Green: Ribosomal binding site and spacer sequence of *Afipia carboxidovorans*
(Siebert *et al.* 2020).
*Blue: *Strep*-tag II (Schmidt and Skerra 2007).

**Table S3:** Protein abundance changes under various conditions. Mixotrophic and chemolithoautotrophic conditions (M, K, and CO) are compared to the heterotrophic reference condition (H). Cultivations were carried out in 50 mL CO-Ox minimal medium under the following conditions: H: 1% [w v^−1^] fructose; M: 1% [w v^−1^] fructose with a gas mixture of 10% CO_2_, 86% H_2_, and 4% O_2_; K: gas mixture of 10% CO_2_, 86% H_2_, and 4% O_2_; CO: gas mixture of 80% CO and 20% synthetic air. H. pseudoflava WT was cultivated in biological quintuplets under each condition (*n* = 5). Proteins were defined as differentially abundant (DA) based on the following thresholds: unique peptides ≥ 2 (DA^1^), p-value ≤ 0.05, q-value ≤ 0.01 (DA^2^), and FC ≤ 0.667 (blue) or ≥ 1.5 (orange) (|log_2_FC| ≥ 0.585). Annotation is based on the corresponding database (CP037867 [chromosome] and CP037868 [megaplasmid pDSM1084]) (Grenz *et al.* 2019) or, alternatively, on manually curated annotations (M.A.) derived from the literature [^a^Grenz *et al.* (2019), ^b^Pohlmann *et al.* (2006), ^c^Siebert *et al.* (2021)], InterPro [^d^Blum *et al.* (2025)], UniProt [^e^The UniProt Consortium (2025)] and BLASTP (Version 2.16.0+) [^f^Altschul *et al.* (1990)].

| Protein (M.A.) | Locus Tag | Description | Log_2_FC(M/H) | Log_2_FC(K/H) | Log_2_FC(CO/H) |
| --- | --- | --- | --- | --- | --- |
| **CO dehydrogenase cluster 1** | | | | | |
| IorA1 | HPF_04350 | Isoquinoline 1-oxidoreductase subunit alpha | 0.16 | 0.13 | 2.59 |
| PucA1 | HPF_04355 | Putative xanthine dehydrogenase subunit A | 0.34 | 0.71 | 5.41 |
| / | HPF_04360 | Hypothetical protein | Not detected | | |
| / | HPF_04365 | Hypothetical protein | 0.63 | 0.91 | 6.44 |
| GlmU1 | HPF_04370 | Bifunctional protein GlmU | 0.77 | 0.93 | 5.66 |
| / (CoxF)^a^ | HPF_04375 | XdhC and CoxI family protein | −0.12 | −0.24 | 3.81 |
| / (CoxE)^a^ | HPF_04380 | VWA domain containing CoxE-like protein | −0.06 | 0.07 | 4.70 |
| / (CoxD)^a^ | HPF_04385 | ATPase family associated with various cellular activities (AAA) | 0.48 | 0.79 | 5.26 |
| / (CoxG)^a^ | HPF_04390 | Carbon monoxide dehydrogenase subunit G | 0.20 | 0.70 | 4.11 |
| CutL1 | HPF_04395 | Carbon monoxide dehydrogenase large chain | 0.29 | 1.25 | 6.65 |
| CutS | HPF_04400 | Carbon monoxide dehydrogenase small chain | 0.49 | 1.28 | 6.61 |
| CutM | HPF_04405 | Carbon monoxide dehydrogenase medium chain | 0.50 | 1.23 | 6.35 |
| RcoM1 | HPF_04410 | Heme-containing CO-sensing transcriptional regulator RcoM1 | −0.61 | Not DA^2^ | 1.78 |
| **CO dehydrogenase cluster 2** | | | | | |
| CoxS | HPF_17415 | Carbon monoxide dehydrogenase small chain | Not DA^2^ | Not DA^2^ | 0.73 |
| CutL2 | HPF_17420 | Carbon monoxide dehydrogenase large chain | −0.41 | −0.51 | 1.09 |
| CoxM | HPF_17425 | Carbon monoxide dehydrogenase medium chain | −0.62 | −0.59 | 1.61 |
| / (CoxD)^a^ | HPF_17430 | AAA domain (dynein-related subfamily) | −0.42 | −0.52 | 1.25 |
| / (CoxE)^a^ | HPF_17435 | VWA domain containing CoxE-like protein | −0.42 | −0.54 | 1.14 |

Table S3: Protein abundance changes under various conditions, continued.

| / (CoxG)^a^ | HPF_17440 | Carbon monoxide dehydrogenase subunit G | 0.36 | Not DA^2^ | 0.78 |
| --- | --- | --- | --- | --- | --- |
| PucA2 (CoxF)^a^ | HPF_17445 | Putative xanthine dehydrogenase subunit A | −0.49 | −0.65 | 1.65 |
| **Hydrogenase cluster** | | | | | |
| HoxK | HPF_01390 | Uptake hydrogenase small subunit precursor | −0.55 | 0.33 | 0.87 |
| HoxG | HPF_01395 | Uptake hydrogenase large subunit | −0.60 | 0.60 | 0.80 |
| / (HupE)^b^ | HPF_01400 | HupE / UreJ protein | Not detected | | |
| HupC1 (HoxZ)^b^ | HPF_01405 | Putative Ni/Fe-hydrogenase *b*-type cytochrome subunit | Not DA^2^ | 1.28 | −0.85 |
| YbaQ | HPF_01410 | Putative HTH-type transcriptional regulator YbaQ | −1.17 | 0.39 | −2.77 |
| HypD (HoxM)^b^ | HPF_01415 | Hydrogenase 2 maturation protease | −0.31 | −0.16 | 2.82 |
| / (HoxL)^b^ | HPF_01420 | HupF/HypC family protein | Not DA^1^ | | |
| HyaE (HoxO)^b^ | HPF_01425 | Hydrogenase-1 operon protein HyaE | Not DA^2^ | Not DA^2^ | 1.67 |
| / (HoxQ)^b^ | HPF_01430 | HupH hydrogenase expression protein | −1.77 | −0.71 | 2.54 |
| Hrb (HoxR)^b^ | HPF_01435 | High molecular weight rubredoxin | Not DA^1^ | | |
| / (HoxT)^b^ | HPF_01440 | Hydrogenase 2-specific chaperone | Not DA^1^ | | |
| / (HoxV)^b^ | HPF_01445 | Hypothetical protein | −1.02 | −0.23 | 1.86 |
| / (HypA)^b^ | HPF_01450 | Hydrogenase nickel incorporation protein HybF/HypA^d^ | −0.31 | Not DA^2^ | 2.63 |
| HypB | HPF_01455 | Hydrogenase isoenzymes nickel incorporation protein HypB | −0.51 | Not DA^2^ | 2.45 |
| HypF1 | HPF_01460 | Hydrogenase maturation protein HypF1 | −1.14 | −0.25 | 1.58 |
| / (HypC)^b^ | HPF_01465 | Hydrogenase expression/formation protein, HupF/HypC^d^ | Not detected | | |
| HypD | HPF_01470 | Hydrogenase expression/formation protein HypD | −0.45 | 0.12 | 2.03 |
| HypE | HPF_01475 | Carbamoyl dehydratase HypE^d^ | −0.79 | Not DA^2^ | 1.63 |
| / | HPF_01480 | Hypothetical protein | −1.44 | Not DA^2^ | 2.20 |
| / (HypX)^b^ | HPF_01485 | Enoyl-CoA hydratase | −0.63 | 0.40 | 3.06 |
| HupR1 (HoxA)^b^ | HPF_01490 | Hydrogenase transcriptional regulatory protein HupR1 | −0.33 | 0.18 | 1.94 |
| HoxF | HPF_01495 | NAD-reducing hydrogenase HoxS subunit alpha | −0.35 | 0.45 | 2.36 |
| HoxU | HPF_01500 | NAD-reducing hydrogenase HoxS subunit gamma | Not DA^2^ | 0.58 | 1.68 |
| HoxY | HPF_01505 | NAD-reducing hydrogenase HoxS subunit delta | −0.69 | Not DA^2^ | 2.93 |
| HoxH | HPF_01510 | NAD-reducing hydrogenase HoxS subunit beta | −0.75 | −0.29 | 2.78 |

Table S3: Protein abundance changes under various conditions, continued.

| / (HoxW)^b^ | HPF_01515 | Endopeptidase^d^ | −0.97 | Not DA^2^ | 2.44 |
| --- | --- | --- | --- | --- | --- |
| / (HoxB)^b^ | HPF_01520 | Periplasmic [NiFeSe] hydrogenase small subunit precursor | −0.17 | 0.63 | 0.77 |
| HydB (HoxC)^b^ | HPF_01525 | Periplasmic [NiFe] hydrogenase large sub-unit precursor | −0.32 | 0.40 | 2.31 |
| / (HoxJ1)^b^ | HPF_01530 | Sensory histidine kinase AtoS | Not DA^2^ | −0.25 | 1.17 |
| FixL1 (HoxJ2)^b^ | HPF_01535 | Sensor protein FixL | Not detected | | |
| **CBB cycle** | | | | | |
| CmpR2 (RbcR)^c^ | HPF_05950 | HTH-type transcriptional activator CmpR | 3.11 | 3.49 | 1.03 |
| CbbL | HPF_05955 | Ribulose bisphosphate carboxylase large chain | 3.28 | 5.08 | 3.33 |
| CbbS1 (CbbS)^c^ | HPF_05960 | Ribulose bisphosphate carboxylase small chain 1 | 1.79 | 3.08 | 1.74 |
| NirQ1 (CbbQ)^f^ | HPF_05965 | Denitrification regulatory protein NirQ | 2.89 | 5.13 | 3.36 |
| / (CbbO)^f^ | HPF_05970 | Von Willebrand factor type A domain protein | 3.06 | 4.84 | 3.67 |
| CmpR3 (CfxR)^c^ | HPF_06180 | HTH-type transcriptional activator CmpR | 1.28 | 0.78 | 0.86 |
| YniC (CbbYP)^c^ | HPF_06185 | 2-deoxyglucose-6-phosphate phosphatase | 1.31 | 4.67 | 3.28 |
| Rpe1 (CbbEP)^c^ | HPF_06190 | Ribulose-phosphate 3-epimerase | 2.61 | 6.04 | 5.42 |
| Fbp1 | HPF_06195 | Fructose-1,6-bisphosphatase class 1 | 0.63 | 3.39 | 2.03 |
| CfxP | HPF_06200 | Phosphoribulokinase, chromosomal | 2.48 | 5.80 | 4.63 |
| TktA1 (CbbTC)^c^ | HPF_06205 | Transketolase 1 | 2.97 | 6.01 | 4.76 |
| Gph1 | HPF_06210 | Phosphoglycolate phosphatase | 2.90 | 5.78 | 1.75 |
| GapA1 | HPF_06215 | Glyceraldehyde-3-phosphate dehydrogenase A | 1.62 | 4.96 | 3.19 |
| Pgk1 | HPF_06220 | Phosphoglycerate kinase | 0.76 | 3.74 | 2.49 |
| Fda1 | HPF_06225 | Fructose-bisphosphate aldolase | 0.74 | 3.83 | 2.09 |
| **Putative isoenzymes for the CBB cycle** | | | | | |
| Fbp2 | HPF_07215 | Fructose-1,6-bisphosphatase class 1 | Not DA^2^ | Not DA^2^ | −0.78 |
| Gph2 | HPF_17815 | Phosphoglycolate phosphatase | Not DA^2^ | Not DA^2^ | −0.69 |
| Rpe2 | HPF_21630 | Ribulose-phosphate 3-epimerase | −1.19 | Not DA^2^ | Not DA^2^ |
| Fda2 | HPF_22300 | Fructose-bisphosphate aldolase | −0.21 | 0.14 | 1.29 |
| Pgk2 | HPF_22510 | Phosphoglycerate kinase | 0.05 | Not DA^2^ | Not DA^2^ |
| GapA2 | HPF_22895 | Glyceraldehyde-3-phosphate dehydrogenase A | 0.40 | 0.47 | −0.49 |
| TktA2 | HPF_22900 | Transketolase 1 | 0.14 | Not DA^2^ | −0.53 |

**Table S3:** Protein abundance changes under various conditions, continued.

| **Entner-Doudoroff pathway** | | | | | |
| --- | --- | --- | --- | --- | --- |
| Eda | HPF_08855 | KHG/KDPG aldolase | Not DA^2^ | −1.60 | −2.24 |
| Edd | HPF_08860 | Phosphogluconate dehydratase | Not DA^2^ | −1.30 | −1.49 |
| Pgl | HPF_08865 | 6-phosphogluconolactonase | Not DA^2^ | −1.56 | −1.95 |
| Zwf | HPF_08870 | Glucose-6-phosphate 1-dehydrogenase | −0.27 | −1.92 | −1.88 |
| **Embden-Meyerhof-Parnas pathway** | | | | | |
| PfkB | HPF_22920 | Putative phosphofructokinase PfkB | Not DA^2^ | 0.20 | −0.92 |
| **Tricarboxylic acid cycle** | | | | | |
| GltA1 | HPF_06915 | Citrate synthase | Not DA^2^ | −0.77 | −0.97 |
| CitZ | HPF_12640 | Citrate synthase 2 | Not DA^2^ | | |
| AcnB | HPF_06960 | Aconitate hydratase 2 | 0.18 | −0.15 | −0.19 |
| AcoA | HPF_07000 | Aconitate hydratase | −0.53 | −0.27 | Not DA^2^ |
| Icd1 | HPF_13460 | Isocitrate dehydrogenase [NADP] | −0.22 | Not DA^2^ | 0.19 |
| Icd2 | HPF_13470 | Isocitrate dehydrogenase [NADP] | −0.31 | −0.45 | −0.46 |
| SucA | HPF_10570 | 2-oxoglutarate dehydrogenase E1 component | −0.57 | −0.85 | −0.53 |
| SucB | HPF_10575 | Dihydrolipoyllysine-residue succinyltransferase component of 2-oxoglutarate dehydrogenase complex | Not DA^2^ | −0.57 | −0.95 |
| Lpd | HPF_10580 | Dihydrolipoyl dehydrogenase | −0.41 | −0.88 | −0.29 |
| LpdA | HPF_10515 | Dihydrolipoyl dehydrogenase | 0.69 | −0.25 | −0.33 |
| SucD1 | HPF_00515 | Succinyl-CoA ligase [ADP-forming] subunit alpha | 0.79 | 1.33 | 1.71 |
| SucD2 | HPF_02590 | Succinyl-CoA ligase [ADP-forming] subunit alpha | 0.10 | 0.31 | 1.01 |
| SucD3 | HPF_22830 | Succinyl-CoA ligase [ADP-forming] subunit alpha | 0.32 | Not DA^2^ | −0.36 |
| SucC1 | HPF_09140 | Succinyl-CoA ligase [ADP-forming] subunit beta | Not DA^2^ | | |
| SucC2 | HPF_22835 | Succinyl-CoA ligase [ADP-forming] subunit beta | 0.25 | Not DA^2^ | −0.87 |
| SdhA | HPF_06930 | Succinate dehydrogenase flavoprotein subunit | 0.02 | −0.55 | −0.66 |
| SdhB1 | HPF_06925 | Succinate dehydrogenase iron-sulfur subunit | Not DA^2^ | −0.30 | −0.90 |
| SdhB2 | HPF_07305 | Succinate dehydrogenase iron-sulfur subunit | −0.97 | −0.38 | 1.70 |
| FumA | HPF_15025 | Fumarate hydratase class I, aerobic | 0.17 | 0.11 | Not DA^2^ |
| FumC | HPF_22980 | Fumarate hydratase class II | −0.33 | 0.39 | 3.33 |
| Mdh1 | HPF_06950 | Malate dehydrogenase | Not DA^2^ | −0.19 | 0.36 |
| **Glyoxylate shunt** | | | | | |
| AceA | HPF_14895 | Isocitrate lyase | 0.40 | 1.09 | 1.73 |
| GlcB | HPF_21795 | Malate synthase G | 1.74 | 1.97 | 0.08 |
| **Glycine cleavage system** | | | | | |
| GcvT | HPF_16545 | Aminomethyltransferase | 2.35 | 2.32 | 1.01 |
| GcvH | HPF_16550 | Glycine cleavage system H protein | 0.72 | 0.98 | 1.10 |
| GcvP | HPF_16555 | Glycine dehydrogenase (decarboxylating) | 1.15 | 1.36 | 0.40 |

**Table S3:** Protein abundance changes under various conditions, continued.

| SdaA | HPF_16585 | l-serine dehydratase 1 | 1.47 | 1.41 | 1.11 |
| --- | --- | --- | --- | --- | --- |
| **Electron transport chain complexes** | | | | | |
| CtaC | HPF_04725 | Cytochrome *c* oxidase subunit 2 precursor | Not DA^2^ | Not DA^2^ | −1.14 |
| CtaD | HPF_04730 | Cytochrome *c* oxidase subunit 1 | Not DA^2^ | Not DA^2^ | −2.57 |
| / | HPF_04735 | Hypothetical protein | Not detected | | |
| CtaG | HPF_04740 | Cytochrome *c* oxidase assembly protein CtaG | −0.98 | −1.03 | −1.95 |
| / | HPF_04745 | Hypothetical protein | Not DA^2^ | −0.34 | Not DA^2^ |
| CtaE1 | HPF_04750 | Cytochrome *c* oxidase subunit 3 | −0.42 | Not DA^2^ | −2.56 |
| / | HPF_04755 | Hypothetical protein | Not detected | | |
| / | HPF_04760 | SURF1 family protein | −0.34 | −0.33 | −0.36 |
| / | HPF_04765 | Hypothetical protein | −0.38 | −0.70 | −1.07 |
| CtaA | HPF_04770 | Heme A synthase | Not DA^2^ | Not DA^2^ | −2.03 |
| CtaB | HPF_04775 | Protoheme IX farnesyltransferase | Not DA^2^ | −1.86 | −2.45 |
| YhjE | HPF_06305 | Inner membrane metabolite transport protein YhjE | 2.60 | 3.27 | 6.28 |
| CyoA | HPF_06310 | Cytochrome *bo_3_* ubiquinol oxidase subunit 2 precursor | 2.73 | 3.56 | 5.09 |
| CyoB | HPF_06315 | Cytochrome *bo_3_* ubiquinol oxidase subunit 1 | 2.15 | 3.39 | 5.87 |
| CyoC | HPF_06320 | Cytochrome *bo_3_* ubiquinol oxidase subunit 3 | 1.23 | 2.13 | 3.45 |
| CyoD | HPF_06325 | Cytochrome *bo_3_* ubiquinol oxidase subunit 4 | Not detected | | |
| / | HPF_06330 | SURF1 family protein | 3.04 | 3.65 | 6.38 |
| / (CcoH)^f^ | HPF_14925 | FixH | 1.93 | 1.86 | 0.15 |
| /  (CcoG)^f^ | HPF_14930 | Quinol dehydrogenase membrane component | 1.53 | 1.74 | 0.70 |
| CcoP | HPF_14935 | *Cbb_3_*-type cytochrome *c* oxidase subunit CcoP | 1.72 | 2.55 | 1.10 |
| FixQ (CcoQ)^f^ | HPF_14940 | *Cbb_3_*-type cytochrome oxidase component FixQ | Not detected | | |
| FixO (CcoO)^f^ | HPF_14945 | Cytochrome *c* oxidase, mono-heme subunit/FixO | 0.60 | 1.89 | 0.55 |
| / (CcoN)^f^ | HPF_14950 | Hypothetical protein | 1.70 | 2.69 | 1.20 |
| / (CcoS)^f^ | HPF_14955 | Cytochrome oxidase maturation protein *cbb_3_*-type | Not detected | | |
| PacS (CcoI)^f^ | HPF_14960 | Putative copper-transporting ATPase PacS | 0.85 | 1.20 | Not DA^2^ |
| PetC | HPF_19600 | Cytochrome *c1* precursor | 0.16 | 0.40 | −0.41 |
| PetB | HPF_19605 | Cytochrome b | Not DA^2^ | 0.26 | Not DA^2^ |
| PetA | HPF_19610 | Ubiquinol-cytochrome *c* reductase iron-sulfur subunit | 0.47 | Not DA^2^ | −0.54 |
| **Further cytochromes** | | | | | |
| HyaC | HPF_02100 | Putative Ni/Fe-hydrogenase 1 *b*-type cytochrome subunit | 0.33 | 0.34 | 1.52 |
| C' | HPF_02105 | Cytochrome *c'* | 0.79 | 1.41 | 2.21 |

**Table S3:** Protein abundance changes under various conditions, continued.

| / | HPF_07360 | Cytochrome *c2* | 1.33 | 0.57 | −1.97 |
| --- | --- | --- | --- | --- | --- |
| / | HPF_14080 | Cytochrome P450 107B1; Monooxygenase or peroxidase activity^d^ | 0.04 | 0.68 | 3.73 |
| BioI | HPF_17070 | Biotin biosynthesis cytochrome P450 | Not DA^2^ | 0.20 | 1.31 |
| / | HPF_17355 | Cytochrome *c-555* precursor | 2.00 | 1.43 | −0.75 |
| AdhB | HPF_21830 | Alcohol dehydrogenase cytochrome *c* subunit precursor | −0.04 | 0.54 | 3.88 |
| Cc2 | HPF_21835 | Cytochrome *c4* precursor | 0.38 | 0.36 | 1.80 |
| / | HPF_22955 | Cytochrome *c*; Cytochrome *c* peroxidase activity^d^ | Not DA^2^ | 0.55 | −1.02 |
| **NADH dehydrogenase complex** | | | | | |
| NdhC | HPF_07495 | NAD(P)H-quinone oxidoreductase subunit 3 | Not DA^1^ | | |
| NuoB | HPF_07500 | NADH-quinone oxidoreductase subunit B | 0.18 | Not DA^2^ | Not DA^2^ |
| NuoC1 | HPF_07505 | NADH-quinone oxidoreductase subunit C1 | Not DA^2^ | Not DA^2^ | −0.35 |
| NuoD | HPF_07510 | NADH-quinone oxidoreductase subunit D | 0.06 | Not DA^2^ | −0.63 |
| Nqo1 | HPF_07515 | NADH-quinone oxidoreductase subunit 2 | 0.32 | Not DA^2^ | −0.39 |
| NuoF1 | HPF_07520 | NADH-quinone oxidoreductase subunit F | Not DA^2^ | Not DA^2^ | −0.26 |
| Nqo2 | HPF_07525 | NADH-quinone oxidoreductase chain 3 | 0.47 | Not DA^2^ | −0.42 |
| NuoH | HPF_07530 | NADH-quinone oxidoreductase subunit H | Not DA^1^ | | |
| NuoI | HPF_07535 | NADH-quinone oxidoreductase subunit I | Not DA^2^ | Not DA^2^ | −0.56 |
| NuoJ | HPF_07540 | NADH-quinone oxidoreductase subunit J | Not DA^1^ | | |
| NuoK | HPF_07545 | NADH-quinone oxidoreductase subunit K | Not detected | | |
| NuoL | HPF_07550 | NADH-quinone oxidoreductase subunit L | Not DA^2^ | | |
| NuoM | HPF_07555 | NADH-quinone oxidoreductase subunit M | Not DA^2^ | Not DA^2^ | −0.70 |
| NuoN | HPF_07560 | NADH-quinone oxidoreductase subunit N | Not DA^1^ | | |
| NuoF2 | HPF_17380 | NADH-quinone oxidoreductase subunit F | −0.22 | Not DA^2^ | 0.98 |
| **Examples of CO-upregulated dehydrogenases** | | | | | |
| QedA | HPF_17540 | Quinoprotein ethanol dehydrogenase precursor | −0.88 | −0.32 | 2.29 |
| LldD | HPF_19300 | l-lactate dehydrogenase [cytochrome] | Not DA^2^ | Not DA^2^ | 1.67 |
| / | HPF_21395 | Quinohemoprotein amine dehydrogenase, beta subunit^d^ | −0.05 | 0.45 | 4.34 |
| QhnDH | HPF_21400 | Quinohemoprotein amine dehydrogenase subunit gamma | Not DA^1^ | | |
| / | HPF_21405 | Quinohemoprotein amine dehydrogenase, gamma subunit maturation protein^d^ | Not DA^2^ | −0.26 | 3.55 |
| / | HPF_21410 | Quinohemoprotein amine dehydrogenase A, alpha subunit, heme binding | Not DA^2^ | 1.16 | 5.07 |
| **Pyrroloquinoline quinone biosynthesis** | | | | | |
| PqqB | HPF_17480 | Coenzyme PQQ synthesis protein B | −1.07 | −0.77 | 4.14 |
| PqqC | HPF_17485 | Pyrroloquinoline-quinone synthase | Not DA^2^ | Not DA^2^ | 1.13 |
| PqqD | HPF_17490 | Coenzyme PQQ synthesis protein D | −0.32 | Not DA^2^ | 1.52 |
| / (PqqE) | HPF_17495 | Pyrroloquinoline quinone biosynthesis protein PqqE | 0.03 | 0.75 | 1.65 |
| PqqE | HPF_22435 | Coenzyme PQQ synthesis protein E | −1.10 | Not DA^2^ | Not DA^2^ |

**Table S3:** Protein abundance changes under various conditions, continued.

| **Stress-associated proteins – General** | | | | | |
| --- | --- | --- | --- | --- | --- |
| / | HPF_00945 | Universal stress protein family protein | Not DA^2^ | −0.56 | −1.18 |
| YhdN1 | HPF_07245 | General stress protein 69 | −0.29 | −0.27 | −0.89 |
| YhdN2 | HPF_08095 | General stress protein 69 | Not DA^2^ | 0.44 | 1.27 |
| / | HPF_14965 | Putative universal stress protein | 0.91 | 2.38 | 3.40 |
|  | HPF_20470 | Universal stress protein family protein | Not DA^2^ | 0.68 | 2.63 |
| **Stress-associated proteins – Signaling** | | | | | |
| PrkC | HPF_05025 | Serine/threonine-protein kinase PrkC | −0.54 | −0.57 | 1.10 |
| PknB | HPF_18260 | Serine/threonine-protein kinase PknB | −0.68 | −0.30 | 1.11 |
| Stp1 | HPF_05030 | Serine/threonine phosphatase Stp | −0.18 | Not DA^2^ | 0.47 |
| Stp2 | HPF_10590 | Serine/threonine phosphatase Stp | 0.45 | 0.30 | 0.95 |
| Stp3 | HPF_22825 | Serine/threonine phosphatase Stp | 0.51 | 0.45 | 0.40 |
| SigG | HPF_10235 | ECF RNA polymerase sigma factor SigG | 0.32 | 0.35 | 2.19 |
| SigL | HPF_11565 | ECF RNA polymerase sigma factor SigL | Not DA^2^ | 0.62 | 2.31 |
| SigR1 | HPF_05360 | ECF RNA polymerase sigma factor SigR | −0.35 | −0.44 | 1.44 |
| SigR2 | HPF_11545 | ECF RNA polymerase sigma factor SigR | −1.96 | −2.17 | −1.87 |
| **Stress-associated proteins – Cell envelope homeostasis** | | | | | |
| CwlK | HPF_00090 | Peptidoglycan l-alanyl-d-glutamate endopeptidase CwlK precursor | 0.24 | −0.01 | 5.57 |
| PgdA1 | HPF_05310 | Peptidoglycan deacetylase | −0.48 | Not DA^2^ | 0.74 |
| PgdA2 | HPF_16275 | Peptidoglycan-N-acetylglucosamine deacetylase | −0.62 | −0.63 | −1.18 |
| UgpB | HPF_19425 | sn-glycerol-3-phosphate-binding periplasmic protein UgpB precursor | −0.20 | 0.47 | 2.44 |
| UgpA1 | HPF_19430 | sn-glycerol-3-phosphate transport system permease protein UgpA | −1.05 | 0.03 | 1.76 |
| AraQ2 (UgpE)^e^ | HPF_19435 | l-arabinose transport system permease protein AraQ | Not DA^1^ | | |
| UgpC3 | HPF_19440 | sn-glycerol-3-phosphate import ATP-binding protein UgpC | Not DA^2^ | 0.37 | 0.71 |
| UgpQ | HPF_19445 | Glycerophosphoryl diester phosphodiesterase | 0.36 | 1.13 | 1.75 |
| SurA1 | HPF_02390 | Chaperone SurA precursor | 0.38 | 0.49 | 2.76 |
| SurA2 | HPF_23025 | Chaperone SurA precursor | 0.03 | Not DA^2^ | −0.14 |
| MucD1 | HPF_18670 | Putative periplasmic serine endoprotease DegP-like precursor | 0.40 | 0.47 | 0.76 |
| MucD2 | HPF_19635 | Putative periplasmic serine endoprotease DegP-like precursor | 0.44 | 0.59 | Not DA^2^ |
| MucD3 | HPF_21845 | Putative periplasmic serine endoprotease DegP-like precursor | 1.82 | 0.82 | −1.30 |
| AprX | HPF_00100 | Serine protease AprX | −0.20 | 0.00 | 3.43 |
| / | HPF_04960 | Putative CtpA-like serine protease | 0.26 | Not DA^2^ | 1.01 |
| Apr1 | HPF_14065 | Subtilisin-like serine protease^d^ | −0.05 | 0.35 | 1.98 |
| Apr2 | HPF_21425 | Subtilisin-like serine protease^d^ | −0.33 | Not DA^2^ | 1.60 |
| DsbB | HPF_13655 | Disulfide bond formation protein B | Not DA^2^ | −2.05 | 0.87 |

**Table S3:** Protein abundance changes under various conditions, continued.

| **Stress-associated proteins – Oxidative stress** | | | | | |
| --- | --- | --- | --- | --- | --- |
| NamA1 | HPF_00245 | NADPH dehydrogenase | 0.62 | Not DA^2^ | −0.69 |
| NamA2 | HPF_03110 | NADPH dehydrogenase | −0.20 | 0.22 | 1.12 |
| / | HPF_20145 | Heme peroxidase | −0.18 | 0.24 | 4.52 |
| KatG | HPF_21440 | Catalase-peroxidase | 0.24 | 0.94 | 2.19 |
| **Further proteins associated with chemolithoautotrophic metabolism** | | | | | |
| RegB | HPF_06335 | Sensor histidine kinase RegB | 0.42 | 0.64 | 2.05 |
| RegA | HPF_06340 | Photosynthetic apparatus regulatory protein RegA | 1.39 | 1.73 | 3.83 |
| CynT | HPF_02395 | Carbonic anhydrase 1 | 0.96 | 1.27 | 5.08 |
| Can1 | HPF_13245 | Carbonic anhydrase 2 | Not DA^2^ | Not DA^2^ | −0.57 |
| Cah | HPF_20765 | Carbonic anhydrase precursor | Not detected | | |
| Can2 | HPF_21140 | Carbonic anhydrase 2 | −0.34 | −0.41 | Not DA^2^ |
| RibBA1 | HPF_05730 | Riboflavin biosynthesis protein RibBA | −0.38 | 0.73 | 3.22 |
| RibBA2 | HPF_08805 | Riboflavin biosynthesis protein RibBA | 0.81 | 0.82 | Not DA^2^ |
| RibD | HPF_16140 | Riboflavin biosynthesis protein RibD | −0.13 | Not DA^2^ | 2.12 |
| **Denitrification** | | | | | |
| NarX | HPF_02325 | Nitrate/nitrite sensor protein NarX | Not DA^2^ | Not DA^2^ | 1.39 |
| NarT | HPF_02360 | Putative nitrate transporter NarT | 0.73 | Not DA^2^ | 1.08 |
| NarK | HPF_02365 | Nitrate/nitrite transporter NarK | 0.76 | 0.98 | 2.63 |
| NarG | HPF_02370 | Respiratory nitrate reductase 1 alpha chain | 0.45 | 0.68 | 3.45 |
| NarH | HPF_02375 | Respiratory nitrate reductase 1 beta chain | 0.85 | 0.92 | 3.06 |
| NarJ | HPF_02380 | Nitrate reductase molybdenum cofactor assembly chaperone NarJ | Not DA^2^ | Not DA^2^ | 1.90 |
| NarV | HPF_02385 | Respiratory nitrate reductase 2 gamma chain | 0.69 | 0.82 | 2.38 |
| NirS1 | HPF_07355 | Nitrite reductase precursor | 2.40 | 1.49 | −1.14 |
| NirS2 | HPF_07395 | Nitrite reductase precursor | 1.58 | Not DA^2^ | −0.61 |
| NorC | HPF_07420 | Nitric oxide reductase subunit C | 1.85 | 0.94 | −1.75 |
| NorB | HPF_07425 | Nitric oxide reductase subunit B | Not DA^1^ | | |
| NirQ2 | HPF_07430 | Denitrification regulatory protein NirQ | 5.32 | 4.25 | 0.52 |
| NosZ | HPF_17320 | Nitrous-oxide reductase precursor | 1.76 | 1.63 | −0.28 |

References

Altschul, S. F., W. Gish, W. Miller, E. W. Myers, and D. J. Lipman. 1990. “Basic Local Alignment Search Tool.” *Journal of Molecular Biology* 215: 403–410.

Blum, M., A. Andreeva, L. C. Florentino, et al. 2025. “InterPro: The Protein Sequence Classification Resource in 2025.” *Nucleic Acids Research* 53: D444–D456.

Grenz, S., P. T. Baumann, C. Rückert, et al. 2019. “Exploiting *Hydrogenophaga pseudoflava* for Aerobic Syngas‐Based Production of Chemicals.” *Metabolic Engineering* 55: 220–230.

Pohlmann, A., W. F. Fricke, F. Reinecke, et al. 2006. “Genome Sequence of the Bioplastic‐Producing ‘*Knallgas*’ Bacterium *Ralstonia eutropha* H16.” *Nature Biotechnology* 24: 1257–1262.

Schäfer, A., A. Tauch, W. Jäger, J. Kalinowski, G. Thierbach, and A. Pühler. 1994. “Small Mobilizable Multi‐Purpose Cloning Vectors Derived From the *Escherichia coli* Plasmids pK18 and pK19: Selection of Defined Deletions in the Chromosome of *Corynebacterium glutamicum*.” *Gene* 145: 69–73.

Schmidt, T. G. M., and A. Skerra. 2007. “The *Strep*‐Tag System for One‐Step Purification and High‐Affinity Detection or Capturing of Proteins.” *Nature Protocols* 2: 1528–1535.

Siebert, D., T. Busche, A. Y. Metz, et al. 2020. “Genetic Engineering of *Oligotropha carboxidovorans* Strain OM5—A Promising Candidate for the Aerobic Utilization of Synthesis Gas.” *ACS Synthetic Biology* 9: 1426–1440.

Siebert, D., B. J. Eikmanns, and B. Blombach. 2021. “Exploiting Aerobic Carboxydotrophic Bacteria for Industrial Biotechnology.” In *One‐Carbon Feedstocks for Sustainable Bioproduction. Advances in Biochemical Engineering/Biotechnology*, edited by A. P. Zeng and N. J. Claassens, vol. 180, 1–32. Springer.

Siebert, D. L., S. Grieshaber, B. Blombach, V. Sieber, and A. Al‐Shameri. 2026. “Biochemical and Mechanistic Insights Into O_2_ Scavenging and Stability of the Soluble Hydrogenase From *Hydrogenophaga pseudoflava*.” *ACS Omega* 11: 1769–1777.

Simon, R., U. Priefer, and A. Pühler. 1983. “A Broad Host Range Mobilization System for *in vivo* Genetic Engineering: Transposon Mutagenesis in Gram‐Negative Bacteria.” *Nature Biotechnology* 1: 784–791.

The UniProt Consortium. 2025. “UniProt: The Universal Protein Knowledgebase in 2025.” *Nucleic Acids Research* 53: D609–D617.

Werner, F., L. S. Schwardmann, D. Siebert, et al. 2023. “Metabolic Engineering of *Corynebacterium glutamicum* for Fatty Alcohol Production From Glucose and Wheat Straw Hydrolysate.” *Biotechnology for Biofuels and Bioproducts* 16: 116.

Zavarzin, G. A., and A. N. Nozhevnikova. 1977. “Aerobic Carboxydobacteria.” *Microbial Ecology* 3: 305–326.
